# The relationship between self, value-based reward, and emotion prioritisation effects

**DOI:** 10.1177/17470218221102887

**Published:** 2022-06-28

**Authors:** Alla Yankouskaya, Gemma Lovett, Jie Sui

**Affiliations:** 1 Department of Psychology, Bournemouth University, Poole, UK; 2 The School of Psychology, University of Aberdeen, Aberdeen, UK

**Keywords:** Self-relevance, value-based reward, positive emotion

## Abstract

People show systematic biases in perception, memory, attention, and decision-making to prioritise information related to self, reward, and positive emotion. A long-standing set of experimental findings points towards putative common properties of these effects. However, the relationship between them remains largely unknown. Here, we addressed this question by assessing and linking these prioritisation effects generated by a common associative matching procedure in three experiments. Self, reward, and positive emotion prioritisation effects were assessed using cluster and shift function analyses to explore and test associations between these effects across individuals. Cluster analysis revealed two distinct patterns of the relationship between the biases. Individuals with faster responses showed a smaller reward and linear positive association between reward and emotion biases. Individuals with slower responses demonstrated a large reward and no association between reward and emotion biases. No evidence of the relationship between self and value-based reward or positive emotion prioritisation effects was found among the clusters. A shift function indicated a partial dominance of high-reward over low-reward distributions at later processing stages in participants with slower but not faster responses. Full stochastic dominance of self-relevance over others and positive over neutral emotion was pertinent to each subgroup of participants. Our findings suggest the independent origin of the self-prioritisation effect. In contrast, commonalities in cognitive mechanisms supporting value-based reward and positive emotion processing are subject to individual differences. These findings add important evidence to a steadily growing research base about the relationship between basic behavioural drivers.

## Introduction

Social cognition collected a vast body of evidence that human perception and decision-making can be affected by the social significance and context in which stimuli appear (Bruch & Feinberg, 2017; Liu & Sui, 2016; Panksepp et al., 2017; Scheller & Sui, 2022; Sui & Humphreys, 2015a). For example, social signals linked to self-relevance, value-based reward, and emotions are typically granted preferential processing in such that people tend to respond faster and more accurately to information containing personal relevance (Sui et al., 2012), higher reward incentives (Yankouskaya, Bührle, et al., 2017), or emotional connotation (Brosch et al., 2013). Although these findings are well documented in the literature, we currently lack detailed accounts of whether and how the properties of these prioritisation effects may relate to each other. Linking these effects is critical for understanding the properties of self-referential processing that carry important implications for mental and affective disorders (Sui & Gu, 2017). Furthermore, it can contribute to the long-standing debates on the relationship between incentive and emotional salience (Berridge, 2018) and how these two powerful drivers of human behaviour are linked to self-referential processes (Madan, 2017).

### Similarity between perceptual effects of self, reward, and emotion

A long-standing set of experimental findings points towards putative common properties of the effects of self-relevance, reward, and emotion on cognitive functioning. For example, all of them can generate robust *facilitation effects* on visual attention selection, such as increasing sensitivity to stimuli and enhancing target detection (Anderson & Yantis, 2013; Bucker & Theeuwes, 2017; Ono & Taniguchi, 2017; Serences & Saproo, 2010; Stolte et al., 2021). Striking evidence arrived from studies measuring the P300, an event-related component of EEG activity that arises when higher-order cognitive operations are related to selective attention and resource allocation. It was found that the amplitude of P300 varied proportionally with the “emotional value” of the stimulus to the perceiver (a stimulus associated with either positive or negative values evoke a larger P300 response compared to neutral stimuli) (Johnston et al., 1986). Reward studies reported similar effects where the P300 showed sensitivity to the magnitude of reward value with a more positive response to a larger than a smaller reward (Sato et al., 2005). The P300 component has been consistently reported in studies on self-referential effects (Gray et al., 2004; Knyazev, 2013; Smigielski et al., 2020). For example, self-relevant relative to control stimuli in an oddball task when self-relevant information (e.g., participants’ middle name, hometown, high school) appeared on the screen as a task-irrelevant stimulus was associated with a large P300 (Gray et al., 2004). It has to be noted that the literature also reports evidence of the modulation of self-reference, reward, and valence in the N1 component which is associated with the early stages of information processing (Citron, 2012; Li et al., 2021; Liu et al., 2016). However, the discussion of the involvement of N1 causes a challenging issue in the field about how early self-relevance may modulate information processing.

Furthermore, meta-analyses of neuroimaging studies showed that neural processes triggered by motivational and self-referential processing may overlap in the cingulate cortex, anterior insula, ventral striatum, dorsolateral, and ventromedial prefrontal cortices (Bartra et al., 2013; Lindquist et al., 2016). Engagement of these regions was observed across a range of reward, emotion, and self-referential tasks, leading to a hypothesis that the same underlying system is responsive to the basic properties of general affect and self-relevance (Heinzel & Northoff, 2014; Northoff & Hayes, 2011).

Another line of evidence indicates that reward, self-relevance, and emotional valence can also affect *perceptual learning*. Particularly, stimuli associated with high reward, self, and emotions are more easily selected and harder to be ignored during perceptual learning. Of direct relevance here is work demonstrating that in a task where participants were required to make anti-saccades for stimuli associated with self, friend, or stranger, suppressing an automatic pro-saccade eye-movement for self-stimuli occurred at a higher cost of voluntary control compared to stimuli related to others (Yankouskaya, Palmer, et al., 2017). Inline, reward studies provided converging evidence that monetary rewards modulated attentional disengagement: signals of high reward could hold attention for longer, even when this was counterproductive for the performance of ongoing tasks (Anderson et al., 2011; Watson et al., 2020). Emotion studies also demonstrated automatic and implicit effects of emotionally valenced relative to neutral stimuli (Astudillo et al., 2018; Tyng et al., 2017) even under direct instructions to ignore the arousing items (Nummenmaa et al., 2006). Similar to self-relevance and reward, emotionally valenced stimuli can capture greater initial attention and inhibit subsequent disengagement from a stimulus location (Fox et al., 2002; Mogg & Bradley, 1999).

Some studies also reported *carryover effects* from initial associations with reward, self, and emotion to subsequent associations formed with the same stimuli. For example, after initial learning stimulus response mapping associated with reward values, the effects of learned associations were carried over to a subsequent visual search task where responses to previously highly rewarded icons were faster than responses to icons associated with a low reward (Krebs et al., 2010; Vartak et al., 2017). Similar effects were found in a study where participants learned associations between shapes and labels (i.e., square—you, triangle—friend, and circle—stranger), denoted personal relevance, and performed a perceptual matching task. However, when the participants were asked to form new associations (e.g., square—friend, triangle—stranger, and circle—you), the performance in a matching task came at the cost of a higher error rate and slower response time (RT) due to a carryover effect from the initial learning (Wang et al., 2016). Carryover effects of emotions that lead to biases in social judgements are well documented in studies on economic decisions (Lerner et al., 2004), attentional control (Fiori & Shuman, 2017), and decision-making (Yates, 2007). The carryover effects indicate that the effects of reward, self-relevance, and emotion can exert automatic influence on cognition.

These similarities between the effects of reward, self-relevance, and emotion on cognitive functions call for a question whether and how these effects relate to each other.

### The relationship between self-relevance, reward, and emotion

Although much experimental work has been done to understand the psychological and neurobiological mechanisms of each effect, these bodies of research have been carried out independently of one another. Research on reward, emotions, and self-reference typically employs topic-specific sets of experimental paradigms and methodological approaches that make linking these effects challenging for exploring the relationship between them. Furthermore, it is well established that individual responses to emotional stimuli, reward incentives, and objects tagged with self-relevance vary greatly across individuals. Exploring individual differences in relation to reward sensitivity (Novak et al., 2016), self-referential processing (Kim & Florack, 2021), and emotionally laden information (Hamann & Canli, 2004) has already provided important insights into the cognitive basis of these effects. However, the individual differences approach to link these effects has not been addressed yet. As such, whether and how these effects relate to each other remains largely unknown.

To date, there is only one theoretical work that systematically investigated neural underpinnings of the relationship between self, reward, and emotion (Northoff, 2016) and empirical evidence from a recent line of research on self-prioritisation (Constable et al., 2021; Stolte et al., 2017; Sui et al., 2015, 2016; Sui & Humphreys, 2015b; Yankouskaya, Humphreys, et al., 2017). Northoff (2016) proposed a model of the self as the most fundamental function of the brain and its associations with basic cognitive functions, such as reward and emotion. Based on the evidence of direct neural interaction between self and emotion from neuroimaging studies, the model predicts a positive parametric dependence between self-specificity and emotion. However, the model is reluctant to make a strong prediction about the relationship between self-relevance and reward.

Notably, since Northoff (2016) model was introduced, new evidence emerged about the relationship between self-relevance, reward, and emotion. The evidence stems from the studies that employed a methodological approach introduced by Sui et al. (2012). This approach uses an experimental procedure in which a basic stimulus (e.g., geometrical shapes, such as circle, triangle, and square) is associatively “tagged” to motivationally significant information (e.g., a word signified personal relevance or reward values, or emotional valence). After the tagging procedure, learned associations are tested in a perceptual matching task where participants are required to indicate whether a displayed shape–label pairing is a matched or mismatched learned pairing. By selecting appropriate stimuli, this methodological approach allows directly linking the effects of self-relevance, reward, and emotion and isolating properties of each effect under the tight control of experimental factors. For example, using this approach, researchers directly tested whether the self-prioritisation effect is associated with the positive valence of self-related stimuli in healthy people and mood disorders (Feldborg et al., 2021; Stolte et al., 2017). However, no correlation was found between self- and emotion-biases (but see Yankouskaya & Sui, 2021). Other studies that tested the relationship between the self and reward using the same experimental paradigm by varying the number of learned associations in each task and the number of displayed exemplars of each category also found no parametric relationship between self- and reward-biases (Sui et al., 2012, 2015; Sui & Humphreys, 2015b; Yankouskaya, Bührle, et al., 2017).

One emerging evidence from the studies above is that self, emotion and reward prioritisation effects have a separate origin (Hobbs et al., 2021; Stolte et al., 2017; Sui et al., 2012, 2015; Sui & Humphreys, 2015a; Yankouskaya, Humphyes et al., 2017). However, given the similarities between these effects on behavioural performance, such a notion warrants asking where the differences lie? Most previous works used the mean as a proxy for inferential statistics. Although this measure has been commonly used as a summary statistic for defining the prioritisation effects, it possesses several limitations, such as it can be misleading when compared distributions are skewed, or experimental manipulations affect the shape of distributions (Balota & Yap, 2011). Moreover, people may use different cognitive strategies during task performance, which cannot be captured by averaging performance across many trials but can be magnified by assessing distribution differences (Rousselet et al., 2017; Rousselet & Wilcox, 2020).

However, there is an important missing piece in previous studies, such as none of them examined the relationship between all three prioritisation effects using a within-subject design. A within-subject design would facilitate the necessary precondition for linking these effects, such as preserving intra-individual variability across all effects. Furthermore, most previous studies into the relationship between these prioritisation effects relaxed the assumption of heterogeneous subpopulations within a study sample. However, it is reasonable to assume that judging stimuli tagged with self-relevance, reward value, and emotion are context-sensitive and may not appeal equally to the entire population.

### The current study

Building on previous research, this study aimed, for the first time, to directly investigate the relationship between the prioritisation effects of self-relevance, value-based reward, and positive emotion. To this end, each participant took part in three separate experiments where we manipulated personal relevance, reward values, and emotional valence using the associative matching procedure described above. This experimental design has three main advantages that we exploited to address this study’s central question: whether and how self, value-based reward, and positive emotion prioritisation effects relate to each other. First, generating the effects of self, reward value, and positive emotion in separate experiments helps deconfound these effects to clarify their relationships. Second, using closely matched experimental designs enables the same metrics to quantify the magnitude of the prioritisation effects. Third, testing and comparing these effects within a single sample permit examining the consistency of these effects and distributional differences between them. To assess the consistency of these effects, we went beyond a common basic statistic summary. We provided detailed information about participants’ performance of how many participants show an effect in the same direction as the group aiming to provide future studies with a good starting point.

We used a novel graphical inferential method called a shift function (Rousselet et al., 2017) to examine distributional differences between the prioritisation effects. The main idea of this method is to describe how one distribution should be rearranged to match another. By quantifying the differences in distributions, we aimed to test whether the relationship between the prioritisation effects can be explained by distributional similarities in the location of these effects. Another way we probe for the relationship between the effects of self, reward value, and positive emotion is by exploring the patterns of individual differences within our sample. Therefore, we explored the structure of our sample using k-means clustering and assessed the relationship within each cluster.

As observed previously in a similar task (Stolte et al., 2017; Yankouskaya, Bührle et al., 2017), we predicted independent processing of self-relevance. Alternatively, we might expect a greater similarity between self- and positive emotion-biases proposed by the neural model (Northoff, 2016). We also anticipated that individual differences might magnify the relationship between the three biases.

## Methods

### Participants

Overall, 61 young healthy adults (age range 19–27 years, *M* = 23.1, *SD* = 3.62, 34 females, 27 males) from Bournemouth University took part in exchange for a partial course credit. The number of participants was estimated using G*Power (V 3.1). To detect a correlation between three variables of interest with effect size = .04, alpha = .033, and power = .80, we need a sample of 49 participants. Our sample exceeds the required number ensuring that the study is well powered.

Exclusion criteria were history of neurodevelopmental conditions, current/past mood disorder, and current/past learning disability. All participants had normal or corrected-to-normal vision. All subjects provided informed consent forms. The study was conducted according to the guidelines of the Declaration of Helsinki and approved by the Research Ethics Committee (REC) Bournemouth University.

### Task, stimuli, and procedure

Participants performed three experiments (labelled here as “Personal Experiment,” “Reward Experiment,” and “Emotion Experiment”). The order of these experiments was randomised across participants. All experiments followed identical procedure, instructions, trial structure and differed only in learned association instructions for personal, reward, or emotion associations, respectively. At the beginning of experimental procedures, six geometric shapes were randomly assigned to labels representing “Me” and “Stranger” (Personal Experiment), “£8” and “£2” (Reward Experiment), “Happy” and “Neutral” (Emotion Experiment). In contrast to previous studies where geometric shapes were associated with emotional expressions depicted in schematic faces (Stolte et al., 2017), we used words representing emotions. Our pilot study suggested that using a word label did not affect the magnitude of emotion prioritisation effects but was beneficial to control for stimuli complexity among the experiments. Furthermore, based on the findings that our Emotion Experiment was designed in healthy participants, prioritising positive emotion is congruent with prioritising self-relevant information (Yankouskaya & Sui, 2021) or reward processing (Pessoa, 2009) to elicit the effects of positive valence to match the prioritisation effects closely.

Instruction for each experiment was displayed on the screen asking participants to remember two shape–label pairings (e.g., Me—circle, Stranger—diamond). Participants were not limited in the amount of time to memorise the associations, but on average, they spent 1–2 min in each experiment.

After the memorising stage, participants had to make fast and accurate judgements on whether displayed shape–label pairings were matched (or mismatched) previously learned associations. In total, 12 practice trials with feedback on accuracy and RT were launched before each experiment. On each trial, a central fixation cross (covering 0.8°×0.8° visual angle) appeared for 500 ms followed by a shape (covering 3.5°×3.5°of visual angle)–label (covering 1.76°/2.52°×1.76°of visual angle) stimulus for 100 ms and a blank interval jittered between 800 and 1500 ms for participants’ response. The left–right positioning of a shape and a label was counterbalanced across trials. The pair either conformed to the written instructions given at the beginning of the experiment (half of the trials) or it was a recombination of a label with a different (mismatched) shape. Participants responded to a shape–label stimulus by pressing the corresponding key (i.e., “n” and “m” keys balanced across participants for responses “correct pairing” and “incorrect pairing”). Feedback on accuracy (words “Correct!” or “Incorrect!”) and RT was provided after each trial in the training. If a participant’s response was slower than 1500 ms, feedback “Too slow!” appeared on the screen and this trial was repeated at the end of practice.

After completing practice trials, participants performed five blocks of 96 trials yielding in total 480 trials in each task (120 trials per condition). In this study, we used three randomisation procedures to minimise the effects of stimuli order within each experiment and experiment order. Specifically, we limited consecutive trials of the same experimental condition to two avoid local structures in the sequences. We randomised and balanced trials within each block. In addition, a randomisation list of experiments was generated for a sample of 60 participants to ensure that each sequence of experiments is equally represented in our data.

**Figure 1.**
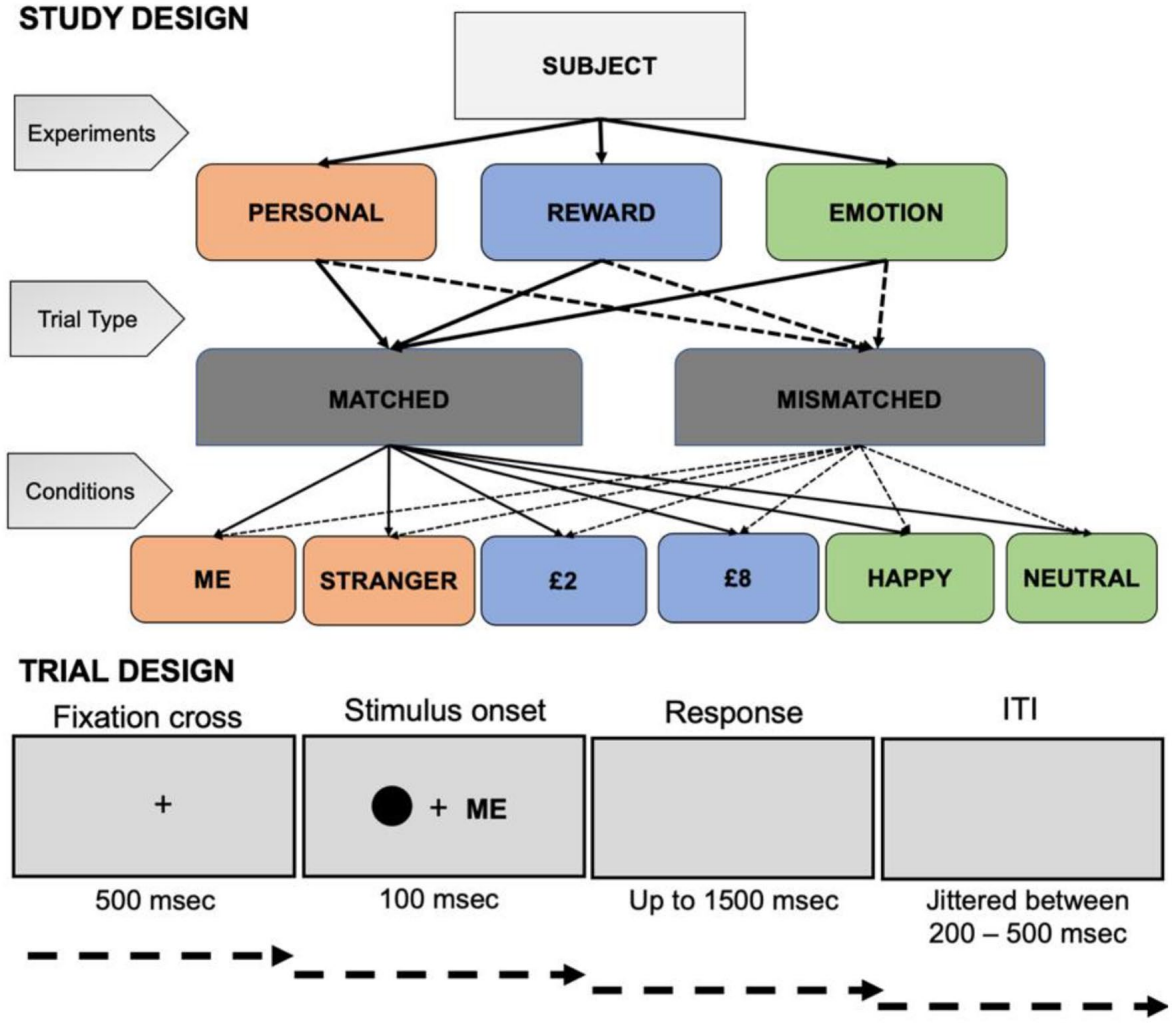
Experimental design in this study.

Participants were required to take breaks between blocks (3 min) and tasks (performed a short distraction task using online jigsaw puzzles, “The boxtrolls puzzle” and “Meet the Pyrenean brown bear” of 25 pieces each [www.hellokids.com]). After each block, participants were provided with feedback on accuracy and averaged RT. If a subject made no response during the blank interval, the feedback “too slow” was appeared on the screen and the stimulus would be presented again by the end of the block (Figure 1).

The experimental design was identical in each experiment. However, in the Reward Experiment, participants were aware that correct responses to matching associations would be rewarded by corresponding bonuses (i.e., £0.08 and £0.02 per trial for the high- and low-reward associations, respectively), and at the end of the experiment, they would receive the total sum of the bonuses based on their performance.

The experiment was run on a PC with a 22″ monitor (1920 × 1080 pixels) at 60 Hz, using E-prime software (Version 2.0) to present the stimuli and record responses.

### Data analysis

#### Measurement reliability analysis

Recent work demonstrated that high measurement error in experiments measuring RT and accuracy could be detrimental to the analysis and the inferences drawn from it (Cooper et al., 2017; Rouder & Haaf, 2019). Therefore, prior to data analysis, we assessed the reliability of our measurements by estimating the internal consistency of RT for each condition in each experiment. Following this line of enquiry and recommended methods for estimating the reliability of cognitive-behavioural measurements, we used a permutation-based split-half approach with 5000 random splits (Parsons, 2020; Parsons et al., 2019). In the split-half method, the data for a measure are split into two halves. The Pearson correlation between these halves with subsequently applied Spearman–Brown (prophecy) correction for the underestimations resulting from splitting the number of observations in half is then calculated as an estimate of the measure’s internal reliability.

#### Descriptive analysis

To gain an overall understanding of the overlap between distributions of prioritisation effects in the Personal, Reward, and Emotion experiments at the groups level, we adopted a bootstrapping procedure (Sui et al., 2012) where mean differences between conditions in Response time (RT) (x) and accuracy (y) for each participant were paired as a single data point (xy) for each experiment. The data sets were then resampled with a replacement but kept the sample size as the number of participants. This procedure was repeated 2000 times, and each resampled set was plotted as a single data point separately for matching and mismatching trials. Following previous studies (Sui et al., 2012), clouds for mismatched trials were defined based on shape category. For example, mismatched “Me” included pairings of a shape associated with “Me” and a label “Stranger”; mismatched “Stranger” included a shape associated with a stranger and a label “Me.”

#### Analysis of prioritisation effects

The proritisation effects were defined as the magnitude of advantages in RT and accuracy and calculated for each participant as follows: [RT_Stranger_–RT_Me_]; [RT_Low reward_–RT_High reward_]; [RT_Neutral_–RT_Happy_]. However, Accuracy advantages were computed in the reversed order (i.e., [Accuracy_Me_–Accuracy_Stranger_]; [Accuracy_High reward_–Accuracy_Low reward_]; and [Accuracy_Happy_–Accuracy_Neutral_]) to display the performance correctly as participants were more accurate for stimuli with higher motivational value.

We analysed these prioritisation effects in two ways. First, we tested whether RT and Accuracy advantages differed across experiments using mixed-effects linear regression models (separate models for RT and Accuracy). For each model, the bias measure (RT or Accuracy advantages) was entered as a dependent variable, and Experiment (Personal, Reward, and Emotion) as a predictor. Individuals were entered as a random effect to account for the random variability among participants. To estimate whether the random effect significantly contributes to the performance, we compared two models with and without the random effect using the Likelihood Ratio Test (LRT). This analysis was performed in JASP (JASP Team 2021; JASP Version 0.16 [Computer software]).

Second, we explored the consistency of self, high reward, and happy emotion prioritisation across the sample. The consistency analysis aimed to answer the following questions: (1) how many participants show an effect in the same direction as the group? (2) how many participants show no effect or an effect in the opposite direction as the group? (3) is there a smooth continuum of effects across participants, or can we identify sub-clusters of participants who appear to behave differently from the rest? This analysis was performed in R using scripts provided in Rousselet et al. (2017).

#### Testing associations between prioritisation effects

First, we tested associations between the prioritisation effects using Pearson correlation. Although correlation has been the most widely used measure of linear dependence between the prioritisation effects, it can underestimate nonlinear contributions if they occur in the data (Smith, 2015). Going beyond previous work, we tested the relationship between self, high reward, and happy emotion prioritisation effects using The mutual information nonparametric test (MINT) implemented in the R package IndepTest (Berrett & Samworth, 2019). This test aims to estimate mutual information between variables based on marginal and joint entropies of these variables using a recently developed efficient entropy estimator (Berrett et al., 2019). The estimator is derived from the nearest neighbours and permits an estimation of critical values by permuting the comparing pairs to mimic the behaviour of the test statistics under H0. If two variables are independent, their joint entropy (denoted as Hxy) equals the sum of their marginal entropies (denoted as Hx + Hy). In the case of some form of dependence between the variables, the joint entropy will be less than the sum of the marginal entropies. Using the MINT, we tested the null hypothesis H0 that the prioritisation effects are independent against the alternative (H1) that they are not independent. We employed the *MINTav* function that performs an independence test without a priori knowledge of either marginal distribution (within the comparing pairs) using permutations and averaging over a range of values of k-neighbours. In addition, the IndepTest package provides a new goodness-of-fit test of normal linear models based on assessing the independence of a vector of covariates and an appropriately defined notion of an error vector (function *MINTregression*). We used this test to examine whether a normal linear model better describes the relationship between compared pairs of RT-biases.

#### Cluster analysis

Next, we implemented an exploratory cluster analysis to identify subgroups of participants with similar patterns of responding to personal relevance, reward, and emotional information. Our cluster variables were RT for matched trials in the three experiments (six variables in total per participant: self, stranger, high reward, low reward, happy, and neutral). We used k-means clustering, the most commonly used unsupervised machine learning algorithm for partitioning a given data set into a set of k groups. The basic idea of the k-means clustering is to classify individuals based on high-intra-class similarity. We employed the standard algorithm for k-clustering, which defines the total within-cluster variation as the sum of squared distances (Euclidean distances) between items and the corresponding centroid (Hartigan & Wong, 1979). Several models were generated and contrasted to determine the most appropriate structure for our data set. Clusters were verified and evaluated by implementing an alternative clustering procedure (agglomerative hierarchical clustering) and cluster validation methods (a Within-Sum-of-Squares plot and a Silhouette plot). They were also quantitatively evaluated on 30 cluster validity criteria within the “NbClust” R package (Charrad et al., 2014). The graphical and clustering methods, validity criteria, and interpretability of each model were compared to determine the final cluster structure. To validate the chosen cluster solution, we used the silhouette coefficient (S*i*) which measures how similar an individual’s performance (*i*) is to the other individuals in their own cluster versus those in the neighbouring cluster. S*i* values range from 1 to −1. A value of S*i* close to 1 indicates that the object is well clustered, while a value close to −1 indicates that the object is poorly clustered, and that assignment to some other cluster would probably improve the overall results (the clustering approach and results are detailed in Supplementary Materials, Note 5).

#### Cluster profiling

We performed cluster profiling by examining distributional differences of self, reward, and emotion prioritisation effects and the relationship between these effects in each cluster. We started with modelling a linear relationship between Experiment and Cluster as our explanatory variables and the magnitude of RT-biases as a dependent variable using a Generalised Linear Mixed Model (GLMM) with Gaussian family and identity link function. Our “full” model used fixed effects (Experiment, Cluster, and interaction between Experiment and Cluster) and participants ID as a random effect grouping factor. The model terms were estimated using a parametric bootstrap procedure with 500 iterations. We used Type II (hierarchical/partially sequential sum of squares) to account for unbalanced sample sizes in our clusters (Langsrud, 2003). To inform our decision on the model, we computed a Bayes factor approximation using the *p*-value calibration introduced by Sellke et al. (2001). The Sellke et al. (2001) *p*-value calibration (Vovk-Sellke maximum *p*-ratio, [VS-MPR]) is a function of the *p*-value, and it is interpreted as the lower bound of the Bayes Factor (favouring *H1–H0*) (Altman & Krzywinski, 2017). We also performed a complementary analysis on means global RT (average reaction time across conditions) for matching trials per cluster to test whether the clusters may differ in the speed of responses. We used a classical mixed analysis of variance with Cluster as a between-subject factor and Experiment as a within-subject factor. All analyses were computed in JASP (JASP Team 2021; JASP Version 0.16 [Computer software]).

Next, we tested associations between self-, reward-, and happy-biases in each cluster using Spearman correlation for accounting for non-normality of data distribution and 1000-iterations bootstrap to estimate the 95% CI.

#### Shift function analysis

To obtain a more detailed understanding of the properties of the prioritisation effects, we tested the location of distributional differences between matched associations in each experiment. Particularly, we were interested whether these differences are pertinent to a specific part of distributions or evenly unfurled. We used a novel graphical inferential method called a shift function (Rousselet et al., 2017). This method has been recently used in cognitive neuroscience to quantify how two distributions differ (Reteig et al., 2018; Rousselet et al., 2016). The main idea of the method is to describe how one distribution should be rearranged to match another: it estimates how and by how much one distribution must be shifted. The shift function plots the pairwise differences between distributions for two conditions (i.e., Condition 1 and Condition 2) along the y-axis for each quantile, as a function of Condition 1 quantiles.

To quantify the differences between distributions at each quantile, we used a hierarchical shift function (HSF) as a powerful alternative to the *t*-test (Rousselet et al., 2017). The HSF was computed as follows. First, the sample quantiles were computed over trials for each participant and each condition. Next, for each participant, Condition 2 quantiles were subtracted from Condition 1 quantiles, and for each quantile, the distribution of differences was subjected to a one-sample test. Next, the quantiles were estimated using the Harrell-Davis quantile estimator (Harrell & Davis, 1982). Finally, a correction for multiple comparisons was applied across the five one-sample tests. Throughout our analyses, we reported *p*-values adjusted for multiple comparisons using Hochberg’s approach (Hochberg, 1988), which guarantees that the probability of at least one false positive will not exceed the nominal level as long as the nominal level is not exceeded for each quantile (Wilcox, 2015). Controlling for multiple comparisons so that the Type I error rate remains around 5% across the nine confidence intervals (this means that the confidence intervals are larger than what they would be if the two distributions were compared at only one quantile) (Rousselet et al., 2017). All calculations were performed using *rogme* R package (Robust Graphical Methods for Group Comparisons [v. 0.2.1]).

## Results

### Pre-processing and data reliability

A data set from one participant was incomplete due to computer failure and was removed from the analyses. The remaining 60 data sets were entered into pre-processing, where we removed outliers defined as RTs faster than 200 ms, which accounted for 1.4% of the overall number of trials. Matching and mismatching trials were analysed separately. The main reason for analysing mismatched trials separately^1^ is that they contain conflicting information involving different cognitive and neural processes during perceptual decision and response selection (Pike & Ryder, 1973).

The (Spearman–Brown corrected) split-half internal consistency in the Personal experiment was *r*_SB_ = 0.85, 95% CI [0.78, 0.90] and *r*_SB_ = 0.80, 95%CI [0.70, 0.87] for “Me” and “Stranger” conditions, respectively. In the Reward experiment, the internal consistency was *r*_SB_ = 0.78, 95% CI [0.68, 0.86] and *r*_SB_ = 0.70, 95%CI [0.56, 0.81] for the high- and low-reward conditions, respectively. In the Emotion experiment, the internal consistency was *r*_SB_ = 0.80, 95% CI [0.70, 0.87] and *r*_SB_ = 0.82, 95%CI [0.72, 0.89] for “Happy” and “Neutral” conditions, respectively. Overall, these results indicated a good reliability (Koo & Li, 2016) of our RT measurement in all experiments .

### Examining self, reward, and emotion prioritisation effects

#### Overlap between the prioritisation effects

**Figure 2.**
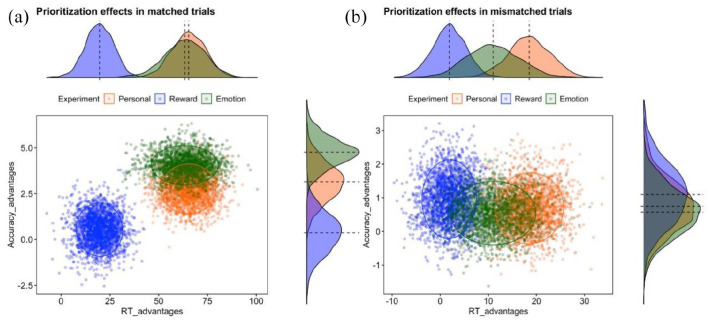
Distributions of prioritisation effects in Personal, Reward, and Emotion Experiments in (a) matching and (b) mismatching trials. The X-axis depicted RT gains. The Y-axis depicted Accuracy gains. Negative values in both axes indicate no prioritisation effects. To visualise the prioritisation effects separately for RT and Accuracy, we provide density plots for corresponding axes.

All three prioritisation effects were significantly greater than zero (Supplementary Material, Note 1). The bootstrapped distributions for matched trials indicated partly overlapping clouds for prioritisation effects in the Personal and Emotion experiments and a separate cloud for the Reward experiment (Figure 2(a)). The clouds for the Personal and Emotion experiments were closer to the upper right corner, indicating greater prioritisation effects in reaction time and accuracy. In contrast, a distribution cloud for the Reward experiment revealed a smaller prioritisation effect in terms of reaction time advantage and near-zero advantage in accuracy performance. Clouds for mismatched trials were largely overlapping between the experiments with no clear gains in accuracy performance. However, there was a tendency for a greater RT advantage in the Personal experiment (Figure 2(b)).

The bootstrap distributions illustrate overlaps among the three types of prioritisation effects. To test these overlaps, we used two mixed-effects linear regression models (separate models for RT- and Accuracy-biases). In the RT model, a fixed effect omnibus test showed a main effect of Experiment, *F*(2, 118) = 13.48, *p* < .001. This effect was driven by significant lower RT advantages in the Reward experiment compared to Personal, MD = 52.70, *t*(118) = −4.58, *p*_holm_ < .001, and Emotion, MD = 50.52, *t*(118) = −4.40, *p*_holm_ < .001, experiments. The mean difference between Personal and Emotion experiments was small, MD = 2.08, *SE* = 11.49, and non-significant, *t*(118) = 0.18, *p*_holm_ = .86. The results of the fixed effect were in line with a complementary Bayesian analysis (Supplementary Material, Note 3).

The variance of the random effect was large (*SD* = 27.67, σ^2^*i* = 765.80, 95% CI [43.31, 1,743.86], ICC = 0.16). The LRT showed that a model with the random effect of individuals explained the performance significantly better compared to a model with a fixed effect only (LRT = 4.29, *df* = 1, *p* = .03). In the Accuracy model, there was a main effect of Experiment, *F*(2, 118) = 22.88, *p* < .001. Post hoc test with Holm’s correction for multiple comparisons indicated that accuracy bias in the Emotion experiment was higher compared to Reward, MD = 3.96, *t*(118) = 3.78, *p*_holm_ < .001, but lower compared to Personal experiment, MD = −7.07, *t*(118) = −6.75, *p*_holm_ < .001. Accuracy in the Reward experiment was lower compared to the Personal experiment, MD = −3.11, *t*(118) = −2.97, *p*_holm_ = .004. The random effect of individuals showed a perfect correlation with the intercept (ICC = 0.00) suggesting that the random effect is redundant (LPR = 0.00).

#### The consistency of prioritisation effects

**Figure 3.**
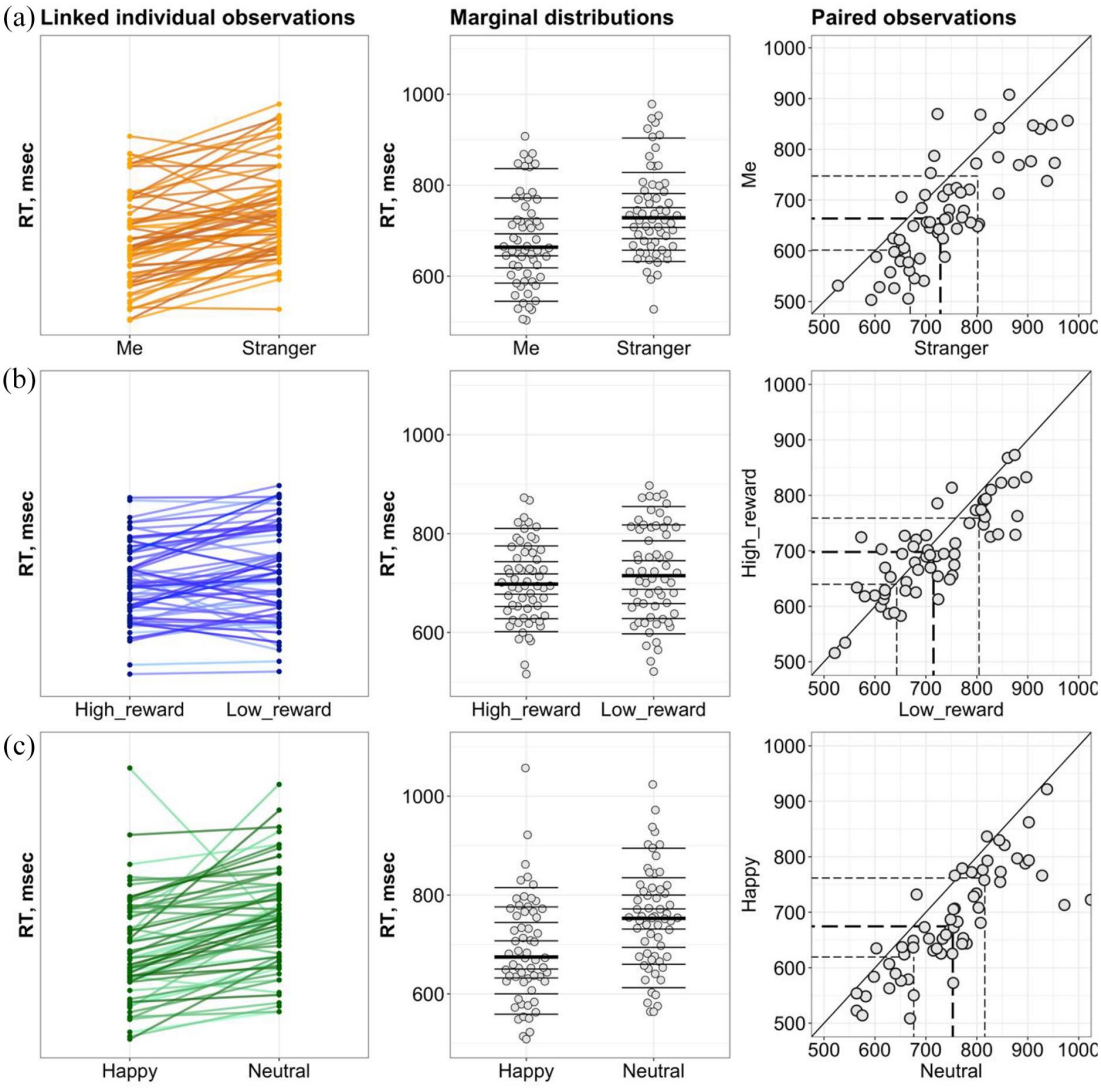
The consistency of the prioritisation effects. (a, b, and c) Personal, Reward, and Emotion experiments, respectively. Left column: the strip charts of linked observations indicating the directionality of the effects among the group. Middle column: marginal distributions for conditions constituting the prioritisation effects where the horizontal lines mark the deciles, with a thicker line for the median. Right column: scatterplots of paired observations where the diagonal line has Slope 1 and Intercept 0 (no prioritisation effect). The dashed lines indicate the quartiles with the thicker line for the median.

The RT mixed-effects linear model suggested a random effect of individual performance. To investigate this effect further, we assessed the consistency of the prioritisation effects. The linked individual observations plots (Figure 3) indicated that in matched trials, 50 out of 60 participants (83.3%) in the Personal experiment and 54 out of 60 (90%) in the Emotion experiment were faster for Me and Happy associations compared to Stranger and Neutral associations, respectively. The marginal distributions plots showed a shift in medians for two dependent distributions in the Personal (Median difference = 58.21, 95% CI [41.21, 74.8]) and Emotion (Median difference = 56.95, 95% CI [38.8, 78.47]) experiment. Only 38 out of 60 (63.3%) participants in the Reward experiment showed the prioritisation effect yielding in a group median shift of 21.35 (95% CI [−0.39, 40.6]). It has to be noted that there was no link between the absence of the prioritisation effect for Me and the magnitude of prioritisation effects for Happy and High reward (Supplementary Material, Note 4).

In mismatched trials, 36 participants (60%) in the Personal experiment, 25 (41.6%) in the Reward experiment, and 28 (46.6%) in the Emotion experiment showed a prioritisation effects. However, paired comparisons reveal no differences between mismatched conditions in each experiment in terms of differences in spread between the conditions and directions of the effects (detailed analyses for mismatched trials are reported in Supplementary Material, Note 4).

#### Linking the prioritisation effects

A correlation analysis indicated that there was no linear association between self- and reward RT-biases (*r* = −.10, *p* = .43, 95% CI [−0.34, 0.15], BF_10_ = 0.319) and between self- and emotion RT-biases (*r* = .097, *p* = .46, 95% CI [−0.16, 0.34], BF_10_ = 0.308). However, there was a moderate correlation between prioritisation effects in the Reward and Emotion experiments (*r* = .47, *p* < .001, 95% CI [0.24, 0.644]). A Bayesian analysis suggested that this correlation is likely to occur 155 times more under the alternative hypothesis (estimated effect size = 0.44, 95% credible interval [0.22, 0.62]). It has to be noted that similar results were obtained when we measured the strength of the relationship between two biases while controlling for the effect of the third one using a partial correlation. Specifically, a correlation between self- and reward RT-biases controlled for emotion bias yielded *r* = −.15, *p* = .21, 95% CI [−0.40, 0.14]; a correlation between self- and emotion-biases controlled for reward-biases showed *r* = .17, *p* = .21, 95% CI [−0.04, 0.35]; a correlation between reward and emotion bias controlled for self-bias was *r* = .48, *p* < .001, 95% CI [0.13, 0.69].

To explore and uncover the association’s underlying patterns, we applied the MINT to RT-biases data. The results indicated that there was no evidence of mutual information between Reward and Self biases (Hx = 5.70, Hy = 5.53, Hxy = 10.96, *p* = .37), Reward and Happy biases (Hx = 5.46, Hy = 5.51, Hxy = 10.11, *p* = .08), and between Self and Happy biases (Hx = 5.70, Hy = 5.51, Hxy = 11.14, *p* = .86). Three separate goodness-of-fit tests of a linear model by testing whether the errors are independent of the covariates in each pair of RT-biases yielded in *p*-values of 1.0, .18, and .95 for Reward–Happy, Self–Reward, and Self–Happy models, respectively. These results indicated that the normal linear model may better fit the data.

### Differential patterns of the relationship between the prioritisation effects

The results of our clustering procedures indicated that a two-cluster model appeared to be the most appropriate accounting for 69.8% of the total variability in the model. The two-cluster solution was verified using agglomerative hierarchical cluster analysis which does not require to pre-specify the number of clusters to be produced. Cluster 1 comprised 23 participants, Cluster 2 consisted of 37 participants. The input variables yielded a silhouette coefficient of 0.41, indicative of fair cluster homogeneity. Detailed cluster analysis is presented in Supplementary Materials, Note 5.

#### Cluster profiling

We calculated RT- and accuracy-biases in each experiment for Cluster 1 and Cluster 2 and tested the effects of Experiment and Clusters on the magnitude of the biases in separate GLMM models. The RT-biases GLMM model showed a main effect of Experiment (χ^2^ = 18.36, *df* = 2, *p* < .001, VS-MPR = 388.08). A main effect of Cluster (χ^2^ = 4.55, *df* = 1, *p* = .03, VS-MPR = 3.27) and interaction between Experiment and Cluster (χ^2^ = 6.28, *df* = 2, *p* = .04, VS-MPR = 2.70) were also significant. The interaction term was driven by higher reward biases in Cluster 1 compared to Cluster 2 (MD = 53.88, *SE* = 17.82, *z* = 3.03, *p*_holm_ = .01, VS-MPR = 6.74). The differences in self- and positive emotion-biases between the clusters were non-significant (all *p*s > .65). In Cluster 1, the differences in the magnitude of biases between experiments were non-significant (all *p*s > .48, see Supplementary Materials, Note 6, Table S5). In Cluster 2, self- and positive emotion-biases were significantly larger compared to reward biases (MD = 73.04, *z* = 5.03, *p*_holm_ < .001, VS-MPR > 100; MD = 59.61, *z* = 4.10, *p*_holm_ < .001, VS-MPR > 100, respectively). The Accuracy-biases GLMM model did not reveal any significant terms (for details, see Supplementary Materials, Note 6).

A complementary analysis on means global reaction time (averaged correct RT for matched trials in each experiment) per cluster to test whether the clusters differ in overall response speed. We found a main effect of cluster, *F*(1, 58) = 4.36, *p* = .04, η_p_^2^ = 0.07). A post hoc test indicated that participants in Cluster 2 were faster compared to Cluster 1, MD = 44.22, *SE* = 21.19, *t*(58) = 2.09, *p* = .04. Replicating this analysis using a Bayesian approach provided support for the difference between clusters (BF_10_ = 18.75).

**Figure 4.**
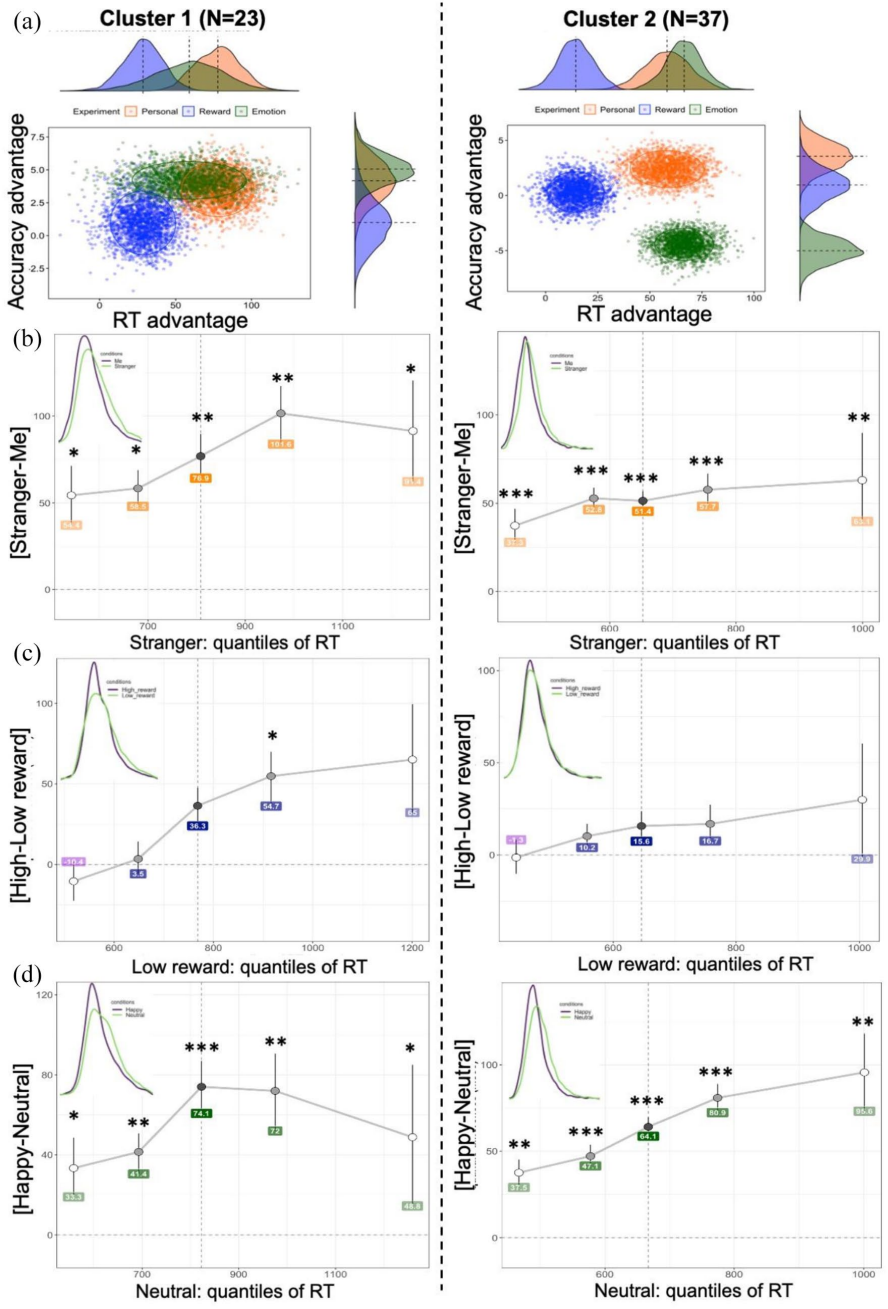
(a) Bootstrapped distributions of prioritisation effects per cluster. (b, c, and d) Shift function in Personal, Reward, and Emotion experiments, respectively. The differences between two matched conditions in each experiment are plotted along the y-axis for each quantile (depicted by dots), as a function of a slower condition quantiles. Quantiles were computed using the Harrell-Davis quantile estimator. For each quantile difference, the vertical line indicates its 95% bootstrap confidence interval (N iterations = 1000). Stars indicate critical *p*-values based on Hochberg’s method after correction for multiple comparison (* < .05, ** < .001, *** < .0001). Kernel density plots in the upper left corners depict the probability density functions of two compared conditions. The vertical dashed line represents the median (.5 quantile).

A correlation analysis showed no association between biases in Cluster 1 (ρ = −0.06, *p* = .77; ρ = 0.22, p = .32; ρ = −0.12, *p* = .59). In Cluster 2, there was a large association between reward and happy biases (ρ = 0.61, 95% CI [0.26, 0.78], *p* < .001) with the VS-MPR indicating that this correlation is 188 times more likely to occur under the alternative hypothesis. To gain an overall understanding about the overlap between bias distributions in each cluster, we plotted prioritisation effects using the bootstrapping procedure (Figure 4(a)). In Cluster 2, the clouds were relatively spaced apart from each other with happy bias showing the lowest accuracy advantage. In contrast, the bias distributions in Cluster 1 were spaced closer to each other with happy bias distributed widely along the RT axis. The reward bias cloud in Cluster 1 showed greater advantages in RT compared to Cluster 2. Self-bias clouds did not differ between the clusters.

##### Analysis of distributional differences

A shift function analysis demonstrated that in both clusters, the differences between “Me” and “Stranger” were positive and significant across all quantiles (Figure 4(b)). Exact *p*-values are reported in Supplementary Materials, Note 7. This systematic effect suggests a complete shift between the two distributions and indicates that self-prioritisation arises as a difference in the location of the distributions starting from early quantiles. However, in Cluster 2, this effect occurred earlier (around 550 ms) and progressed uniformly across all quantiles yielding an average effect of 50 ms. In contrast, Cluster 1 showed a 100 ms delay in the occurrence of self-prioritisation with a progressively larger effect size from Quantile 1 to 4. The shift function in each cluster was relatively consistent with only two participants in Cluster 1 (8.7%) and three participants in Cluster 2 (8.1%) who did not show complete or partial stochastic dominance of “Me” over “Stranger.” The results suggest a full stochastic dominance of responses to the self over responses to a stranger among the clusters. However, the clusters differ in the location of the largest differences defining the prioritisation effect.

In the Reward experiment, a shift function and the kernel density estimate indicate late differences between high- and low-reward distributions (Figure 4(c)). This finding was evident in Cluster 1, where the differences survived correction for multiple comparisons only at the fourth quantile. In contrast, the distribution differences in Cluster 2 were very weak throughout all quantiles. An additional analysis testing the distributions of bootstrapped estimates confirmed the results of the shift function showing that the 90% highest-density interval crossed the zero line at each quantile in Cluster 2 (Supplementary Materials, Note 7, Figure S11). Individual shift functions showed that four participants (17.4%) in Cluster 1 and eight participants (21.6%) in Cluster 2 had all quantile differences less than zero. The results indicate that the reward prioritisation effect is generated at later processing stages in both clusters. However, participants in Cluster 1 showed a greater advantage of high-reward value over low-reward value, while in Cluster 2, the shift between distributions was small and statistically not significant.

In the Emotion experiment, the shift between distributions for Happy and Neutral conditions was fairly similar among the clusters (Figure 4(d)). The shift was positive and significant at all quantiles indicating stochastic dominance Happy over Neutral emotion. Only one participant (4.3%) in Cluster 1 and two participants (5.4%) in Cluster 2 showed all quantile differences less than zero suggesting that the shift is consistent within each cluster. The kernel density estimates suggest almost uniform marginal shifts to the right for the slower condition (Neutral) among the clusters. Interestingly, in Cluster 2, the largest differences between distributions of happy and neutral responses fail at the latest quantiles indicating that positive emotion prioritisation in faster participants can be associated with the later stage of processing (e.g., response selection). In contrast, Cluster 1 showed considerable differences around the middle quantile with greater uncertainty of the shift at the later quantile.

The results of our shift function analyses indicated three main findings: (1) the prioritisation effects show a central tendency in participants with slower responses, while in participants with faster responses, these effects tend to enlarge towards the distribution tail; (2) disregard of the location of the main distributional differences, self and positive emotion prioritisation showed full stochastic dominance over less favourable options (stranger and neutral emotion) among the sample; (3) participants with slower responses generate significant value-based prioritisation effect, while participants with faster responses did not show a systematic effect of high-reward over low-reward conditions.

## Discussion

We set out to examine whether biased responses to self-relevant, reward, and emotional information are linked. Using matching experimental designs, we measured the biases in RT and accuracy of responding to Me/Happy/£8 relative to the less favourable options (Stranger/Neutral/£2). The article seeks to investigate whether and how the magnitude of these biases relate to each other. To achieve this aim, we, first, provided a detailed analysis of the facilitation effects of self-relevance, high reward, and positive emotion on performance to understand the consistency of these biases and their magnitude. Next, we examined an association between the magnitude of these biases and tested whether their relationship is pertinent to individual differences in processing speed. Finally, we also explored whether the individual differences depend on how the two levels in each experiment (i.e., Me vs Stranger, High vs Low reward, Happy vs Neutral) relate to each other.

In line with previous studies, our results demonstrated that items tagged with self-relevance, positive emotion, and high reward exerted a potent influence on stimulus processing, such as faster and more accurate RT (Sui et al., 2016; Sui & Humphreys, 2015b; Yankouskaya, Humphreys, et al., 2017). We also replicated previous findings that the magnitude of self-bias was larger than reward bias (Yankouskaya, Bührle, et al., 2017) but did not differ from positive emotion bias (Stolte et al., 2017). The consistency with previous findings and the reliability of our behavioural measures suggest that these effects are unlikely to be due to a measurement error.

### A group-level perspective on the relationship between self, reward, and emotion prioritisation effects

#### The relationship between self-bias and motivational biases

In highlighting the effects of self-relevance, reward, and positive emotion on perceptual matching, the current research extends previous work on this topic (Hobbs et al., 2021; Northoff & Hayes, 2011; Qian et al., 2020). Our results provide striking evidence that linear or nonparametric mutual information models cannot capture associations between the magnitude of self-bias and motivational biases. Moreover, the absence of the relationship between self and two motivational factors, such as reward and positive emotion, within subgroups of participants with similar performance further support the claim that the facilitation effect of self on information processing cannot be reduced to reward or positive emotion processing (Sui & Gu, 2017; Sui & Humphreys, 2015a). One possible explanation of this finding is that when self, reward, or emotion are processed independently within the associative matching task, their context-specific information triggers distinct neural networks. Recent fMRI studies support this explanation by demonstrating differential involvements of neural networks for processing self-biases compared to positive emotion biases (Yankouskaya & Sui, 2021) or reward biases (Yankouskaya, Humphreys, et al., 2017 It has to be noted that temporary changes in state mood, such as negative mood induction, may inhibit self-prioritisation (Sui et al., 2016). This is consistent with the evidence in mental health. A recent study showed reduced self-prioritisation in sub-clinically anxious individuals (Feldborg et al., 2021; but see Hobbs et al., 2021). Furthermore, there is an indication of the relationship between self- and positive emotion-biases in a selected non-clinical sample with strict control of mood and anxiety symptoms (Yankouskaya & Sui, 2021). These findings indicate that independence may be subject to functional changes, and from this perspective, they are in line with the hypothesis predicting a parametric relationship between self- and emotion-bias (Northoff, 2016).

#### The relationship between motivational biases

Using a within-subject experimental design allowed us to capture important regularities in the relationship between reward and emotion biases. First, we found a significant positive association between the prioritisation of reward and happy emotion. It has to be noted that, the parametric relationship between reward and positive emotion biases was consistent only in 61.7% of individuals, while the rest of the participants (Cluster 1) showed no sign of associations between them. This is the first demonstration of individual differences in the relationship between reward and emotion prioritisation effects to the best of our knowledge. Assessing individual differences in affective information processing is not new in the field (Gohm & Clore, 2002; Pessoa, 2009). However, considering them as a possible factor determining the extent to which the association between the biases occurs may reconcile puzzling inconsistency in previous attempts to link positive emotion and value-based reward (for review, see Chiew & Braver, 2011; Sander & Nummenmaa, 2021).

Second, the relationship between the prioritisation of value-based reward and positive emotion generated by the associative matching procedure (Sui et al., 2012) is likely to occur linearly. The results of the MINT regression analysis provided strong evidence against a nonparametric nature of the relationship. If confirmed in future studies, exploring properties of this linear relationship may shed light on common and dissociable influences of reward incentives and positive emotion on cognitive functioning (Chiew & Braver, 2011). Interestingly, some evidence indicates that reward incentives may promote a positive mood (Meloy et al., 2006). However, the evidence is relatively sparse, and the mechanisms underlying the relationship between value-based reward and positive emotion remain largely unknown.

Several theoretical accounts conceptualised the relationship between emotion and value-based reward, considering emotion as an emergent property of motivationally driven neural activity (Buck, 2000; Panksepp et al., 1998) or linking motivational salience and appraised relevance (Sander et al., 2018). However, empirical supports for these accounts received inconsistent results partly from methodological limitations, such as using different experimental paradigms for assessing reward and emotion biases (Park et al., 2018, 2019). In this study, we addressed methodological limitations of previous work by (1) employing identical experimental procedures to generate the prioritisation effects; (2) exerting tight control over stimuli; and (3) unlinking the biases by assigning them to separate experiments. Our findings indicate that the association between reward and emotion biases emerges in the larger proportion of individuals without explicitly linking these effects.

### Individual differences in the relationship between the prioritisation effects

Our exploratory cluster analyses provide further insights on the relationship between self, reward, and emotion prioritisation effects. First, while the global RT of the first and second clusters was just over 44 s apart, Bayesian analysis provided support for this difference, indicating that participants in Cluster 2 were generally faster compared to individuals in Cluster 1. Second, slower participants (Cluster 1) showed no associations between self, reward, and emotion prioritisation effects. In contrast, faster participants (Cluster 2) manifested a strong correlation between reward and emotion biases. Third, the speed of responding did not affect the magnitude of self- and positive emotion-biases—we did not find evidence of the differences between the clusters in the magnitude of either self or emotion prioritisation. However, Cluster 1 showed significantly higher reward bias than Cluster 2, indicating that the overall speed of responding may affect reward processing. This finding is in line with our previous work demonstrating that the rate of evidence accumulation and responding speed are functions of reward and subject of individual differences that could explain a large proportion of variance in responding to high- and low-reward values (Yankouskaya, Bührle, et al., 2017). Future studies manipulating perceptual properties of the stimuli and reward values may shed more light on these findings.

One prominent view referred to as a dual-process theory distinguishes between slower and faster responses in terms of “intuitive/faster” and “deliberative/slower” processes (Achtziger & Alós-Ferrer, 2014; Kahneman, 2011). Decisions produced by an intuitive process that are quick but prone to certain biases tend to have shorter RTs. In contrast, a deliberative process that carefully weighs the available options is executed slower. The key distinction between these two processes is that deliberative processes rely on features of a choice while intuitive processes are less sensitive to choice details (Krajbich et al., 2015 but see Pennycook et al., 2016). The dual-process approach can provide a working explanation of the cluster differences in reward processing. Notably, participants adopting the intuitive processes might be less sensitive to reward values and respond with the same speed. In contrast, individuals adopting deliberative processes could be more biased by higher reward value. An alternative explanation of the low-reward bias in Cluster 2 is that the amount of money at stake was probably not challenging enough for participants in Cluster 2, and reflection on reward could lead them to disengage from the pursuit and attainment of reward (Zedelius et al., 2013). Although both explanations need experimental testing, they may direct further research. It has to be noted that slower and faster responses were not compromised by trading off speed and accuracy. In this study, participants were instructed to respond fast and accurately, ensuring that we did not induce the response strategy experimentally. Moreover, the relative simplicity of the task and stimulus difficulty kept constant across all experiments minimised the need for participants to adjust their decision-making strategy. Therefore, individual differences in adopting the intuitive or deliberative processes are likely to reflect the preferred type of reasoning.

Our GLMM models on means RT advantages showed no significant differences between self- and positive emotion-biases within and between the clusters. However, going beyond the measures of central tendency revealed interesting findings; here, we used a shift function to characterise how the prioritisation effects come about by assessing how one distribution should be rearranged to match another one. The self and positive emotion prioritisation effects in Cluster 1 were more prominent at two middle quantiles (0.5 and 0.75), yielding the largest differences between two distributions constituting these effects. In Cluster 2, prioritisation of self was driven by an almost uniform shift across all quantiles, while the effect of positive emotion was determined by gradually increasing differences between happy and neutral towards the tail of the distributions. A good illustration of these findings is presented in Figure 4.

Importantly, in each cluster, a shift function suggested full stochastic dominance of “self” over “stranger” or “happy emotion” over “neutral” disregard of the location of the largest differences between them. This finding indicates that self and emotion prioritisation may penetrate cognitive processes deeper than previously thought. For example, a commonly reported finding is that self-prioritisation is underpinned by differences in the rate of information uptake and characterised by a higher drift-diffusion rate compared to other shape-category pairings (e.g., for friend and stranger) (Golubickis et al., 2017; Hu et al., 2020). However, the high drift rate alone cannot explain the full stochastic dominance observed in this study. One reason why previous drift-diffusion models could not capture this effect is that a typical drift-diffusion model relies on the assumption of constant stopping boundaries (boundaries between two choices—“matched” vs “mismatched”) over time. This assumption might not be held on a trial-to-trial basis when matched, and mismatched trials presented randomly. It is worth mentioning that choosing fixed and freely varied parameters in the drift-diffusion approach is somewhat arbitrary and depends on hypothesis testing. When the duration of all extra-decisional parts of the RT and a drift rate were allowed to vary between conditions, the effects of self were observed in the later processing time (Yankouskaya, Bührle, et al., 2017). Furthermore, in contrast to drift-diffusion models that consider an overall task performance, a shift function defines distributional differences between two conditions constituting the effects, thus, providing information about the relative strength of the effect across the entire distribution.

It has to be noted that the utility of understanding the relationship between self-relevance, reward, and emotion processing may be extended beyond basic research to clinical research. These are motivated, in part, by well-documented evidence about impaired self-referential processing, adverse reward sensitivity, and emotion disorders associated with many mental health conditions (Borsini et al., 2020; Feldborg et al., 2021; Hobbs et al., 2021; McIvor et al., 2021). However, their influence on cognition generally has not been explicitly considered in relation to one another because decompounding these effects is a challenge for exploring and clarifying these relationships (Chiew & Braver, 2011). This study demonstrated the unlinked effects of self-relevance, value-based reward, and emotion using matched experimental tasks. A future consideration is to link these effects and explore their relationship by examining the effects of these manipulations have on each other. For example, manipulating the “relatedness” of reward or emotional valence to the self or linking the prioritisation effects by presenting a task-irrelevant distractor and measuring the interference effects can help to examine whether a common basis of self is manifested in the prioritisation effects in a domain-independent way.

### Limitations

We used a k-means clustering procedure for partitioning our sample. Although we performed several complementary analyses to inform our clustering solution and carried out validation procedures, this method possesses a number of limitations, such as sensitivity to extreme values and the absence of standard algorithms for selecting the most appropriate number of clusters (Steinley & Brusco, 2011). Moreover, there is no consensus in the literature for sample size-to-variable ratio in cluster analyses (Steinley, 2006). Therefore, we used a general rule of thumb, such as 5 × (2^k), where k represents the number of variables (Formann, 1988) which indicates that the present sample size of 60 is adequate for our cluster analysis. However, replication of the cluster structure with a large sample is needed to support our findings further.

In this study, we did not use the pre-screening procedure to control for depressive, anxious, and mixed symptomatology. Recent study (Hobbs et al., 2021) showed that depression symptoms could not predict the magnitude of emotion-bias. However, controlling for mood could add more precision to our GLMM models.

Previous research using cross-cultural comparison demonstrated the effects of cultural differences on the magnitude of self-prioritisation (Jiang et al., 2019; Jiang & Sui, 2022; Sparks et al., 2016; Sui & Han, 2007). Although our sample was relatively homogeneous in terms of cultural variations, generalising our findings to diverse populations should be taken with caution.

## Conclusion

Our results provide no evidence of the relationship between self and value-based reward or positive emotion prioritisation effects suggesting the independent origin of self-bias. The relationship between value-based reward and positive emotion prioritisation is subject to individual differences. Individuals with faster responses showed a smaller reward and linear positive association between reward and emotion biases. Individuals with slower responses demonstrated a large reward and no association between reward and emotion biases. Individual differences do not determine a partial order between self and positive emotion distributions. Both prioritisation effects were generated by full stochastic dominance of more favourable stimuli (self or happy emotion) over less favourable stimuli (stranger or neutral emotion). In contrast, the value-based prioritisation effect in participants with slower responses was generated by a partial stochastic dominance of high-reward value at the later processing stages. Individuals with faster responses showed no significant differences between high- and low-reward distributions.

## Supplemental Material

sj-docx-1-qjp-10.1177_17470218221102887 – Supplemental material for The relationship between self, value-based reward, and emotion prioritisation effects Click here for additional data file. Supplemental material, sj-docx-1-qjp-10.1177_17470218221102887 for The relationship between self, value-based reward, and emotion prioritisation effects by Alla Yankouskaya, Gemma Lovett and Jie Sui in Quarterly Journal of Experimental Psychology

## Notes

1 Previous studies using the associative matching task consistently reported longer response latencies for mismatching than matching trials (Schäfer et al., 2017).
