# Supplementary material for: The relationship between self, value-based reward, and emotion prioritisation effects
Source: Q J Exp Psychol (Hove). 2022 Jun 28;76(4):942–60. doi: 10.1177/17470218221102887 (PMC10031635; doi:10.1177/17470218221102887)
Supplement: sj-docx-1-qjp-10.1177_17470218221102887 – Supplemental material for The relationship between self, value-based reward, and emotion prioritisation effects [file sj-docx-1-qjp-10.1177_17470218221102887.docx]

**Supplementary Material**

The relationship between self, value-based reward and emotion prioritisation effects

(Yankouskaya, Lovett & Sui)

**Note 1**

***Testing whether self-, reward- and emotion-prioritization effects in RT were significantly greater than zero***

These effects were reliably greater than zero for matching trials (t(59) = 7.63, p<.001, Cohen’s d = 0.98; t(59) = 2.75, p=.004, Cohen’s d = 0.35; t(59) = 6.18, p < .001, Cohen’s d = 0.79 in the Personal, Reward, Emotion experiments respectively). A Bayesian analysis support this finding by providing decisive evidence for the alternative hypothesis in the Personal and Emotion experiments (BF_10_ >100) and moderate evidence in the Reward experiment (BF_10_ = 8.67, estimated effect size = 0.339, 95%CI [0.093, 0.597]). A prioritization effect in the Personal experiment was also found in mismatching trials (t(59) = 4.243, p<.001, Cohen’s d = 0.58, BF_10_ >100). No evidence for prioritization effects in mismatching trials was found in Reward and Emotion experiments (t(59)=0.21, p=0.6, Cohen’s d=0.06, BF_10_ = 0.218; t(59) = 2.06, p=.043, Cohen’s d=0.26, BF_10_ = 1.98).

**Note 2.**

***Prioritization effect in accuracy performance***

Accuracy performance was defined as percept correct responses for each condition in matched and mismatched trials per participant. The magnitude for accuracy advantage was calculated as absolute differences between accuracy scores (i.e. [“Stranger”-“Me”], [“Low reward”-“High reward”], [“Neutral”-“Happy”]) for matched and mismatched trials (Figure S1).

Figure S1. The magnitude for accuracy advantage in matched and mismatched trials per experiment. The error bars represent +/-SEM.

A one-way repeated measures analysis of variance (ANOVA) was carried out on accuracy advantages in the Personal, Reward and Emotion experiments for matched and mismatched trials separately.

For matched trials, there was a main effect of experiment (F(2, 118) = 5.85, p = .004, η^2^ = 0.09, BF10 = 10.90. Post Hoc comparisons with Holm adjustment for multiple comparisons showed that the main effect was driven by greater accuracy biases in the Emotion compared to reward experiment (MD = 3.53, t (59) = 3.47, p_Holm_ = .003, BF_10_ = 27.08). The differences between Personal and Emotion, and Personal and Reward experiments were non-significant (MD = 1.37, t(59) = 1.50, p = .14, BF_10_ = 0.41; MD = 2.17, t(59) = 1.84, p = .14, BF_10_ = 0.68 respectively).

For mismatched trials, there was no effect of experiment on accuracy biases (F(2, 118) = .41, BF_10_ = 0.08).

**Note 3**

***The results of a Bayesian analysis on RT-biases***

A one-way repeated-measures Bayesian ANOVA was performed on matched trials to test the effects of Experiment (Personal, Reward, Emotion) on the RT biases. The results of a Bayesian analysis indicated that the data were 509 times more likely under the model that included Experiment as a predictor, compared to the null model. Post Hoc comparisons of the magnitude of prioritization effects between Personal vs Reward and Emotion vs Reward experiments revealed posterior odds of 56.0 and 609.19 against the null hypothesis which indicated decisive evidence in favour of the alternative hypothesis. When comparing prioritization effects between Personal and Emotion experiments there was no evidence for the alternative hypothesis (posterior odds = 0.084). For mismatched trials, a Bayesian analysis provided only anecdotal evidence for the main effect of Experiment (the data were 1.93 times more likely under the model that included Experiment as a predictor, compared to the null model).

**Note 4**

***The absence of prioritisation effect for self and related effects***

In the Personal Experiment, 16.7% of our participants showed no prioritisation effect of self. We performed a detailed examination of whether the absence of self-prioritisation is linked to a lack of prioritisation of happy emotion or reward. The data are displayed below:

| ID | Self-bias | Reward-bias | Emotion-bias | Experiment order• |
| --- | --- | --- | --- | --- |
| 3 | -11.65 | 23.39 | 86.38 | P-E-R |
| 21 | -4.14 | 5.18 | 33.59 | P-R-E |
| 22 | -44.12 | 17.57 | 34.99 | R-E-P |
| 26 | -70.64 | 2.65 | -7.74 | E-P-R |
| 30 | -53.77 | 49.43 | 80.43 | E-R-P |
| 42 | -43.81 | -9.35 | 38.79 | R-P-E |
| 47 | -61.20 | 146.90 | 27.95 | P-E-R |
| 54 | -146.73 | 111.60 | 83.53 | E-P-R |
| 58 | -70.64 | 2.65 | 10.20 | R-P-E |
| 60 | -7.38 | 15.67 | 65.54 | P-E-R |
| Mean | -51.41 | 36.57 | 45.36 |  |
| SD | 41.88 | 51.99 | 32.36 |  |

•P, R, E represent Personal, Reward and Emotion Experiment respectively

Plotting the data does not reveal a pattern

***Consistency of prioritization effects in mismatched trials***

In mismatched trials, 36 participants in the Personal experiment, 25 in the Reward experiment and 28 in the Emotion experiment showed a prioritization effect for a shape associated with high saliency. However, paired comparisons (Fig. S2) reveal no differences between mismatched conditions in each experiment in terms of differences in spread between the conditions and directions of the effects. The distribution of within-participant differences (see Paired observations plots in Fig. S2) revealed similar patterns across the experiments.


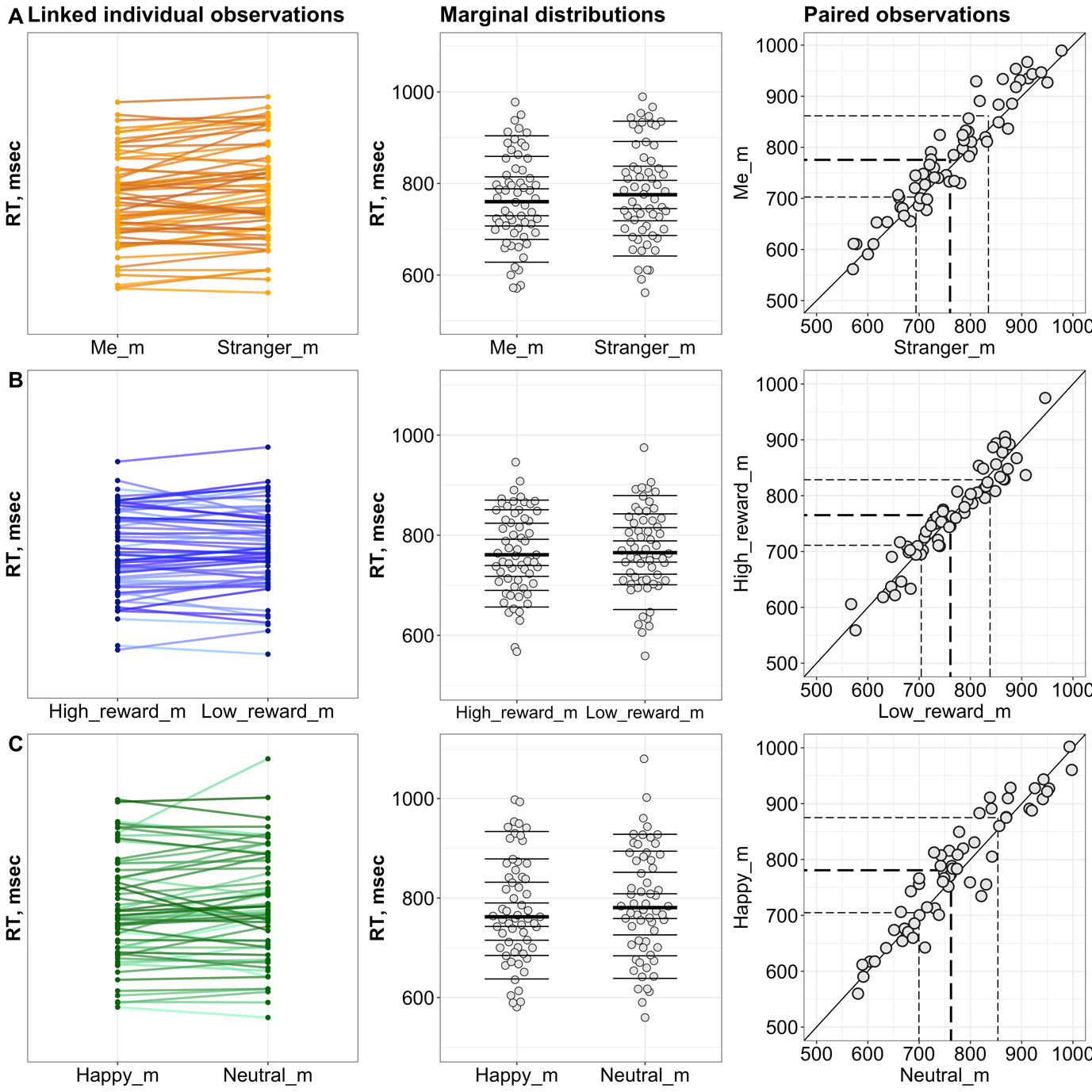


*Figure S2.* Mismatched trials. Panels A, B, C correspond to Personal, Reward and Emotion experiments respectively. The stripcharts (left column) display the linked observations between conditions indicating the directionality of the effects. Middle column: marginal distributions for conditions constituting the prioritization effects. Horizontal lines mark the deciles, with a thicker line for the median. The right column presents a scatterplot of paired observations where the diagonal line has slope 1 and intercept 0 (no prioritization effect).

**Note 5**

***Cluster analysis***

*Distance measurements*. The Euclidian distances between pairs of observations were computed using R base function *dist()* [*stats* package] (*Fig. S3*). The measure of Euclidian distance was chosen in order to cluster together observations with high (low) values of features. Before computing the distances, the variables were scaled to have standard deviation one and mean zero.


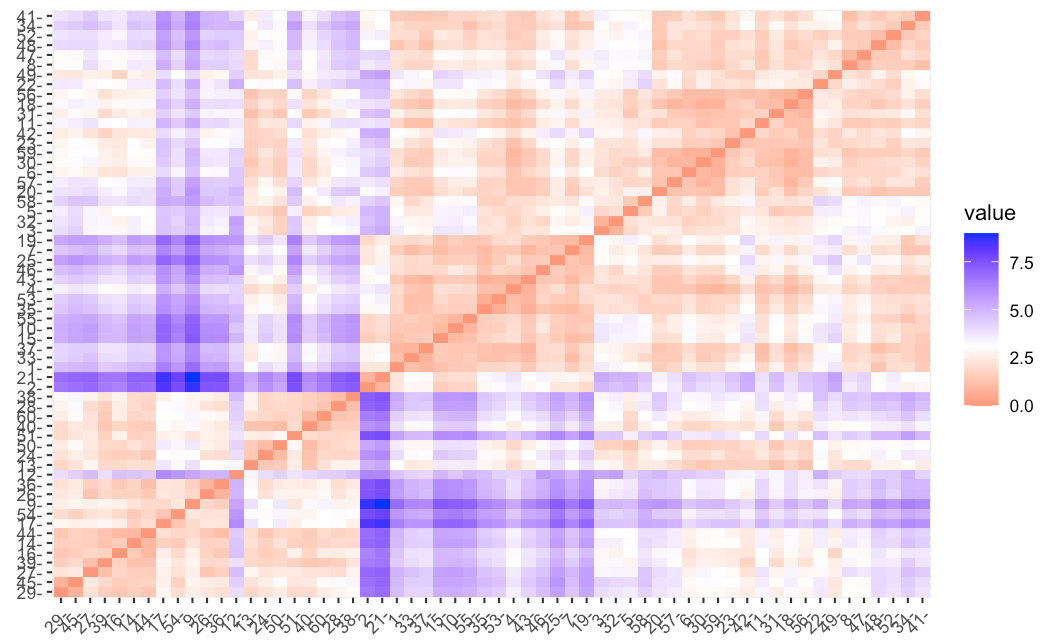


*Figure S3.* Euclidian distances between pairs of observations. Colour coding: red represents high similarity, blue represents low similarity. The color level is proportional to the value of the dissimilarity between observations: pure red if *dist*(xi, xj ) = 0 and pure blue if *dist*(xi, xj ) = 1. Objects belonging to the same cluster are displayed in consecutive order.

*Determining the number of clusters.* To determine the number of clusters, we employed *NbClust*() function from NbClust R package (Charrad et al., 2014). NbClust simultaneously computes 30 indices for determining the relevant number of clusters and proposes the best clustering solution from different results obtained by varying all combinations of the number of clusters, distance measures, and clustering methods (Charrad et al., 2014). The results of NbClust among all indices:

12 proposed 2 as the best number of clusters

6 proposed 3 as the best number of clusters

1 proposed 5 as the best number of clusters

3 proposed 6 as the best number of clusters

2 proposed 9 as the best number of clusters

In addition, two graphical methods were used to confirm the results of the NbClust analysis. First, we applied the elbow method to decide the number of clusters based on the explained variation and choosing the point where the curve visibly bends. Second, we used the silhouette plot which displays a measure of how close each point in one cluster is to points in the neighbouring clusters (Figure S4).


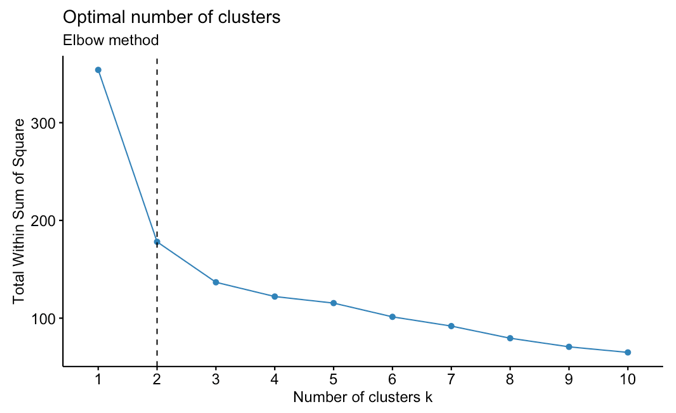

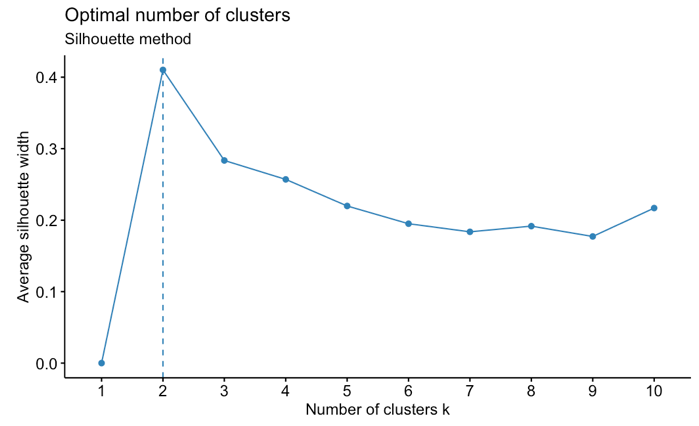


*Figure S4*. Graphical methods to determine the optimal number of cluster in the present study (the Elbow plot on the left and the Silhouette plot).

*Clustering approach*. A K-means clustering algorithm (MacQueen, 1967) was applied to determine the presence of k groups based on 60 observations. This approach allows us to classify participants such that individuals within the same cluster are as similar as possible (i.e., high intra-class similarity), whereas participants from different clusters are characterised as having low inter-class similarity. K-means clustering was performed using

the standard R function for k-means clustering is *kmeans*() [*stats* package]. To inform further our decision on the number of clusters, we also computed clustering solutions with 3, 4 and 5 clusters (Fig. S5).


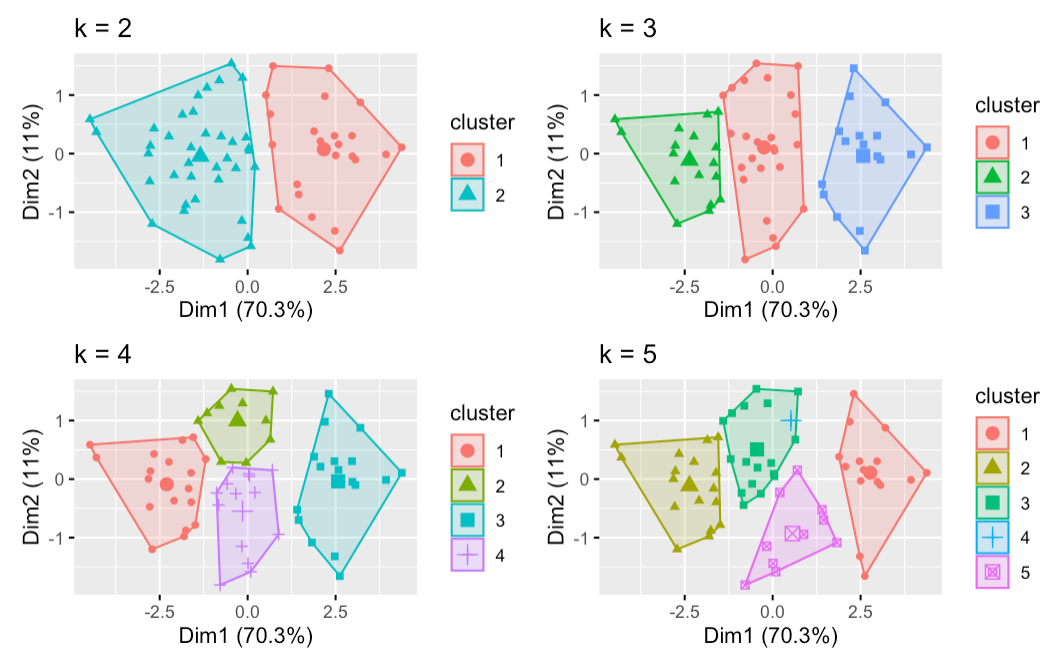


*Figure S5*. Clustering solutions with k=2 to k=5. The starting point was specified as 50 to obtain a more stable result for each solution with the number of iterations of 1000. The figure was plotted using the *factorextra* R package ().

The clustering solutions displayed in Fig. S5 indicate that we can discharge the 5-cluster solution as the *kmeans*() was not able to define 5 clusters. Therefore, we focused on comparing the 2-4 clustering solutions. The results of this comparison indicated that the 2-cluster solution yielded the largest difference between clusters (Fig. S6, Table S1).


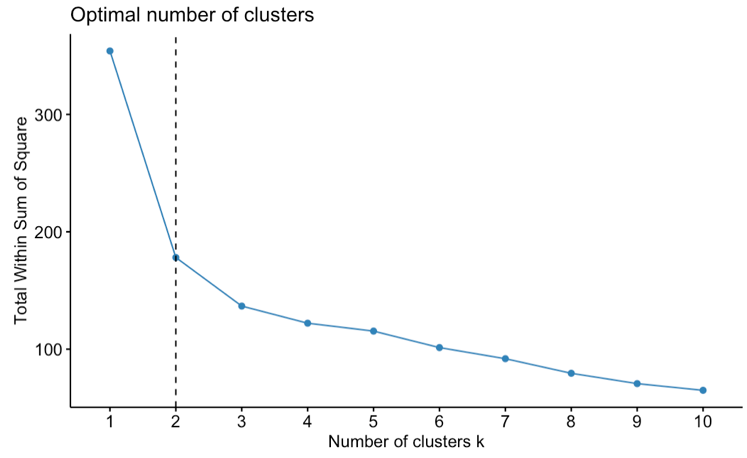


*Figure S6*. Plot of the WSS (within-sum of squares) representing the variance within the clusters. The location of a bend (knee) in the plot is considered as an indicator of the appropriate number of clusters.

Table S1. The results of clustering solutions in the present study

| Solution | Cluster sizes | Between SS/ Total SS (%) |
| --- | --- | --- |
| 2-clusters | 23, 37 | 69.8 |
| 3-clusters | 25, 17, 18 | 65.6 |
| 4-clusters | 18, 10, 18, 14 | 49.6 |

The final solution of two clusters is presented in Fig. S7 using *fviz_cluster*() function from *factorextra* R package.


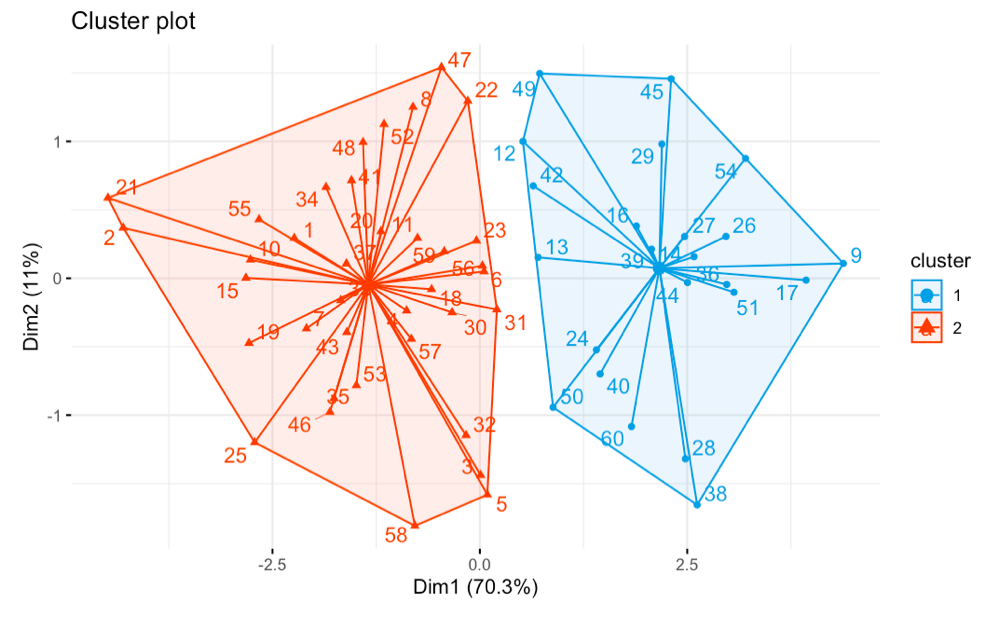


Figure S7. The 2-cluster solution in the present study. The observations are represented by points with numbers identifying individual data sets. The centres represent cluster means.

*Verifying the 2-cluster solution*. We used agglomerative hierarchical cluster analysis (a-HCA) as an alternative approach to k-means clustering for grouping individuals based on their similarity. In contrast to partitioning clustering, hierarchical clustering does not require to pre-specify the number of clusters to be produced. With agglomerative clustering, in which each observation is initially considered as a cluster of its own (leaf), the most similar clusters are successively merged until there is one single big cluster (root) (Fig. S8).

After applying the agglomerative cluster analysis to our data, we assessed whether the distances (i.e., heights) in the dendrogram reflect the original distances accurately by computing the correlation between the cophenetic distances and the original distances. If the clustering is valid, the linking of objects in the cluster tree should have a strong correlation with the distances between objects in the original distance matrix.

To compute the cophenetic distances we used the *cophenetic*() function in R. The correlation coefficient (r=0.73) indicated a good relationship between the cophenetic distances and the original distances.


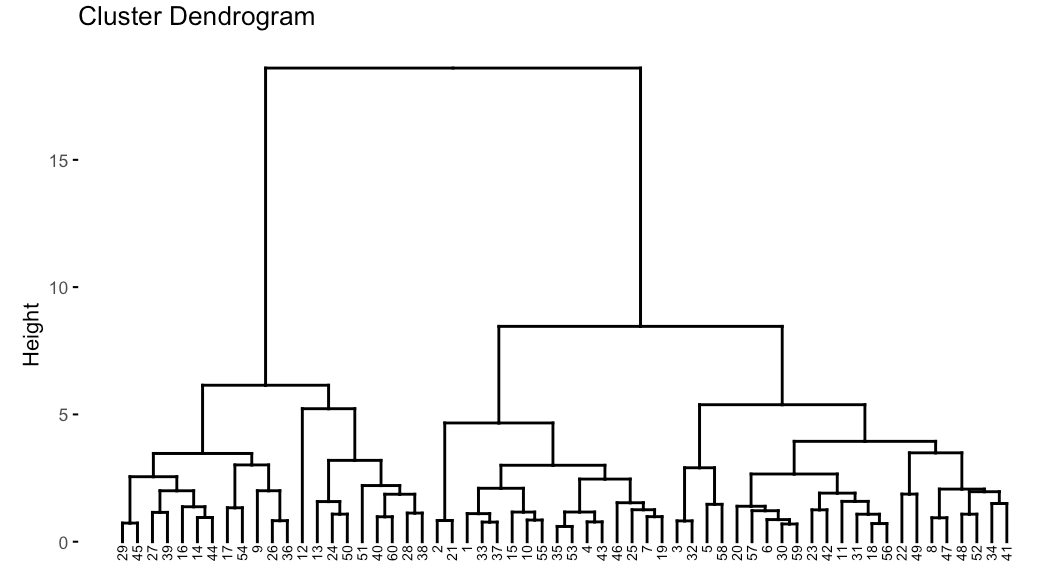


*Figure S8*. Dendrogram representing the results of hierarchical clustering in the present study using Ward’s minimum variance method

*Cluster validation*. To validate the clusters in the present study, we used the silhouette coefficient (S) measures how similar an individual is to the other individuals in its own cluster versus those in the neighbour cluster. S values range from 1 to -1. A value of S close to 1 indicates that the individual is similar to the other individuals in their group. In contrast, a value of S close to -1 indicates that an individual is poorly clustered, and that assignment to some other cluster would probably improve the overall results.

To draw silhouette coefficients, we used the function *fviz_silhouette*() [*factoextra* package]. This analysis is summarised in Fig. S9. The average silhouette width of each cluster is: 0.37 and 0.43 respectively for cluster 1 and cluster 2. Overall, these results indicate that the individuals are relatively well-clustered.


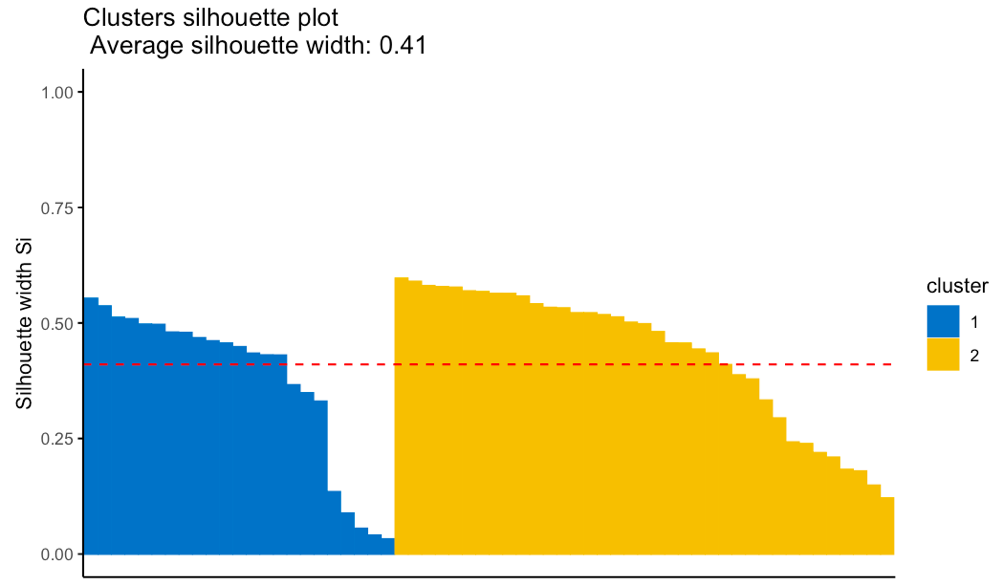


*Figure S9*. Validation of two clusters. The Y-axis represents the silhouette coefficients (the silhouette width of observations). The X-axis represents individuals within each cluster.

**Note 6**

***Cluster profiling: a Generalised Linear Mixed Model (GLMM)***

*RT-biases GLMM*. The results of the RT-biases GLMM model are detailed in Tables S2-S5 below.

| **Table S2. ANOVA Summary** | | | | | | | | | |
| --- | --- | --- | --- | --- | --- | --- | --- | --- | --- |
| **Effect** | | **df** | | **ChiSq** | | **p** | | **VS-MPR*** | |
| Experiment |  | 2 |  | 18.356 |  | < .001 |  | 388.076 |  |
| Cluster |  | 1 |  | 4.546 |  | 0.033 |  | 3.268 |  |
| Experiment ✻  Cluster |  | 2 |  | 6.276 |  | 0.043 |  | 2.703 |  |
|  | | | | | | | | | |
|  | | | | | | | | | |
| *Note.*  Generalized linear mixed model with gaussian family and identity link function. | | | | | | | | | |
| *Note.*  Model terms tested with likelihood ratio tests method. | | | | | | | | | |
|  | | | | | | | | | |
| *Note.*  The following variable is used as a random effects grouping factor: 'ID'. | | | | | | | | | |
| *Note.*  Type III Sum of Squares | | | | | | | | | |
| * Vovk-Sellke Maximum *p* -Ratio: Based on a two-sided *p* -value, the maximum possible odds in favor of H₁ over H₀ equals 1/(-e *p* log(*p* )) for *p* ≤ .37 (Sellke, Bayarri, & Berger, 2001). | | | | | | | | | |

| **Table S3. Fixed Effects Estimates** | | | | | | | | | |
| --- | --- | --- | --- | --- | --- | --- | --- | --- | --- |
| **Term** | | **Estimate** | | **SE** | | **t** | |  | |
| Intercept |  | 50.886 |  | 5.787 |  | 8.793 |  |  |  |
| Experiment (1) |  | 15.506 |  | 6.771 |  | 2.290 |  |  |  |
| Experiment (2) |  | -29.640 |  | 6.771 |  | -4.377 |  |  |  |
| Cluster (1) |  | 12.365 |  | 5.787 |  | 2.137 |  |  |  |
| Experiment (1) ✻  Cluster (1) |  | 0.115 |  | 6.771 |  | 0.017 |  |  |  |
| Experiment (2) ✻  Cluster (1) |  | 14.577 |  | 6.771 |  | 2.153 |  |  |  |
|  | | | | | | | | | |
| *Note.*  The intercept corresponds to the (unweighted) grand mean; for each factor with k levels, k - 1 parameters are estimated with sum contrast coding. Consequently, the estimates cannot be directly mapped to factor levels. Use estimated marginal means for obtaining estimates for each factor level/design cell or their differences. | | | | | | | | | |

| **Table S4. Estimated Marginal Means** | | | | | | | | | | | | | |
| --- | --- | --- | --- | --- | --- | --- | --- | --- | --- | --- | --- | --- | --- |
|  | | | | | | | | | | **95% CI** | | | |
| **Row** | | **Experiment** | | **Cluster** | | **Estimate** | | **SE** | | **Lower** | | **Upper** | |
| 1 |  | Happy_bias |  | 1 |  | 78.872 |  | 13.990 |  | 51.453 |  | 106.292 |  |
| 2 |  | Reward_bias |  | 1 |  | 48.188 |  | 13.990 |  | 20.769 |  | 75.608 |  |
| 3 |  | Self_bias |  | 1 |  | 62.693 |  | 13.990 |  | 35.274 |  | 90.113 |  |
| 4 |  | Happy_bias |  | 2 |  | 53.911 |  | 11.030 |  | 32.293 |  | 75.530 |  |
| 5 |  | Reward_bias |  | 2 |  | -5.696 |  | 11.030 |  | -27.314 |  | 15.922 |  |
| 6 |  | Self_bias |  | 2 |  | 67.346 |  | 11.030 |  | 45.728 |  | 88.965 |  |
|  | | | | | | | | | | | | | |
| *Note.*  Results are on the response scale. | | | | | | | | | | | | | |

| **Table S5. Contrasts** | | | | | | | | | | | | | |
| --- | --- | --- | --- | --- | --- | --- | --- | --- | --- | --- | --- | --- | --- |
|  | | **Estimate** | | **SE** | | **df** | | **z** | | **p†** | | **VS-MPR** | |
| Contrast (Happy1>Happy2*) |  | 24.961 |  | 17.815 |  | ∞ |  | 1.401 |  | 0.645 |  | 1.000 |  |
| Contrast (Reward1>Reward2) |  | 53.884 |  | 17.815 |  | ∞ |  | 3.025 |  | 0.015 |  | 5.859 |  |
| Contrast (Self1>Self2) |  | -4.653 |  | 17.815 |  | ∞ |  | -0.261 |  | 1.000 |  | 1.000 |  |
| Contrast (Happy1>Reward1) |  | 30.684 |  | 18.420 |  | ∞ |  | 1.666 |  | 0.479 |  | 1.000 |  |
| Contrast (Self1>Reward1) |  | 14.505 |  | 18.420 |  | ∞ |  | 0.787 |  | 1.000 |  | 1.000 |  |
| Contrast (Happy2>Reward2) |  | 59.607 |  | 14.523 |  | ∞ |  | 4.104 |  | < .001 |  | 158.668 |  |
| Contrast (Self2>Reward2) |  | 73.042 |  | 14.523 |  | ∞ |  | 5.029 |  | < .001 |  | 7508.676 |  |
| Contrast (Happy1>Self1) |  | 16.179 |  | 18.420 |  | ∞ |  | 0.878 |  | 1.000 |  | 1.000 |  |
|  | | | | | | | | | | | | | |
| *Note.*  Results are on the response scale. | | | | | | | | | | | | | |
| † P-values are adjusted using Holm adjustment. | | | | | | | | | | | | | |
| *Numbers 1 and 2 denote Cluster 1 and Cluster2 | | | | | | | | | | | | | |

The raincloud plots (Figure S10) provide detailed visualisation of the raw data, the distribution of the data, and key summary.

| ***Cluster: 1*** | ***Cluster: 2*** |
| --- | --- |
| 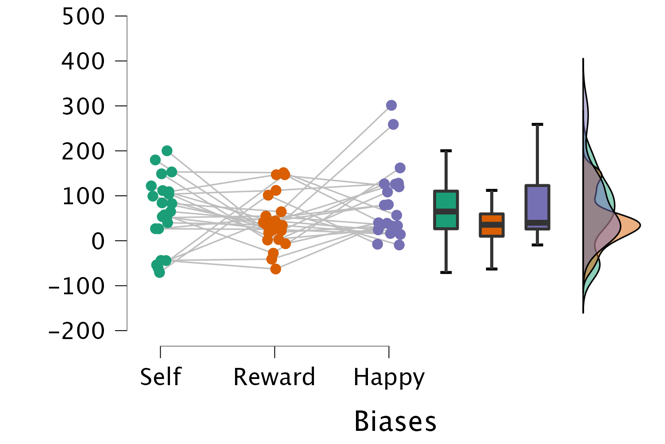 | 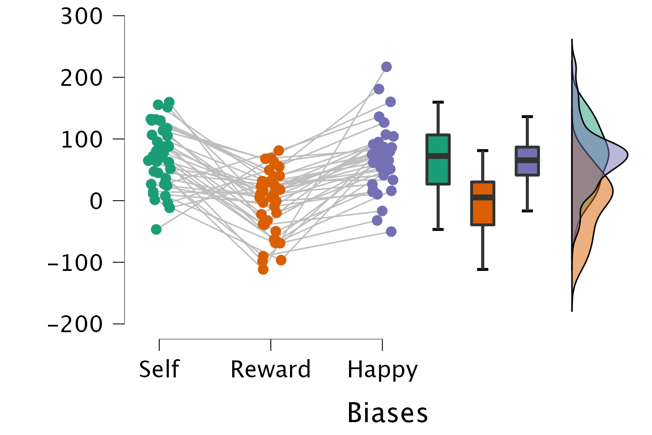 |

*Figure S10.* Linked means RT biases in each cluster. Boxplots display the median and the 95% CI for each median.

*Accuracy-biases GLMM*. The results of the Accuracy-biases GLMM model are detailed in Tables S6-S8 below.

| **Table S6. ANOVA Summary** | | | | | | | | | |
| --- | --- | --- | --- | --- | --- | --- | --- | --- | --- |
| **Effect** | | **df** | | **ChiSq** | | **p** | | **VS-MPR*** | |
| Experiment |  | 2 |  | 5.396 |  | 0.067 |  | 2.025 |  |
| Cluster |  | 1 |  | 2.349 |  | 0.125 |  | 1.413 |  |
| Experiment ✻  Cluster |  | 2 |  | 3.277 |  | 0.194 |  | 1.156 |  |
|  | | | | | | | | | |
| Note.  Generalized linear mixed model with gaussian family and identity link function. | | | | | | | | | |
| Note.  Model terms tested with likelihood ratio tests method. | | | | | | | | | |
| Note.  The following variable is used as a random effects grouping factor: 'ID'. | | | | | | | | | |
| Note.  Type III Sum of Squares | | | | | | | | | |
| * Vovk-Sellke Maximum p -Ratio: Based on a two-sided p -value, the maximum possible odds in favor of H₁ over H₀ equals 1/(-e p log(p )) for p ≤ .37 (Sellke, Bayarri, & Berger, 2001). | | | | | | | | | |

| **Table S7. Fixed Effects Estimates** | | | | | | | | | |
| --- | --- | --- | --- | --- | --- | --- | --- | --- | --- |
| **Term** | | **Estimate** | | **SE** | | **t** | |  | |
| Intercept |  | 2.008 |  | 0.616 |  | 3.259 |  |  |  |
| Experiment (1) |  | 0.352 |  | 0.589 |  | 0.598 |  |  |  |
| Experiment (2) |  | -1.313 |  | 0.589 |  | -2.231 |  |  |  |
| Cluster (1) |  | 0.937 |  | 0.616 |  | 1.522 |  |  |  |
| Experiment (1) ✻  Cluster (1) |  | 1.040 |  | 0.589 |  | 1.766 |  |  |  |
| Experiment (2) ✻  Cluster (1) |  | -0.675 |  | 0.589 |  | -1.147 |  |  |  |
|  | | | | | | | | | |
| Note.  The intercept corresponds to the (unweighted) grand mean; for each factor with k levels, k - 1 parameters are estimated with sum contrast coding. Consequently, the estimates cannot be directly mapped to factor levels. Use estimated marginal means for obtaining estimates for each factor level/design cell or their differences. | | | | | | | | | |
|  | | | | | | | | | |

| **Table S8. Estimated Marginal Means** | | | | | | | | | | | |
| --- | --- | --- | --- | --- | --- | --- | --- | --- | --- | --- | --- |
|  | | | | | | | | **95% CI** | | | |
| **Experiment** | | **Cluster** | | **Estimate** | | **SE** | | **Lower** | | **Upper** | |
| Happy_bias |  | 1 |  | 4.337 |  | 1.338 |  | 1.714 |  | 6.959 |  |
| Reward_bias |  | 1 |  | 0.956 |  | 1.338 |  | -1.666 |  | 3.579 |  |
| Self_bias |  | 1 |  | 3.542 |  | 1.338 |  | 0.919 |  | 6.165 |  |
| Happy_bias |  | 2 |  | 0.383 |  | 1.055 |  | -1.685 |  | 2.451 |  |
| Reward_bias |  | 2 |  | 0.432 |  | 1.055 |  | -1.636 |  | 2.500 |  |
| Self_bias |  | 2 |  | 2.396 |  | 1.055 |  | 0.328 |  | 4.464 |  |
|  | | | | | | | | | | | |
| Note.  Results are on the response scale.  ***Complementary analysis***  We performed a complementary analysis on means global response time (average reaction time across conditions ) for matching trials per cluster to test whether the clusters may differ in speed of responses. We used a classical mixed analysis of variance with Cluster as a between-subject factor and Experiment as a within-subject factor (see Complementary Tables 1 - 3 for main effects and Post Hoc comparisons. The results of a Bayesian analysis is reported in Complementary Table 4.   \| **Complementary analysis Table 1. Within Subjects Effects** \| \| \| \| \| \| \| \| \| \| \| \| \| \| \| \| \| --- \| --- \| --- \| --- \| --- \| --- \| --- \| --- \| --- \| --- \| --- \| --- \| --- \| --- \| --- \| --- \| \|  \|  \|  \|  \|  \|  \|  \|  \|  \|  \|  \|  \|  \|  \|  \|  \| \|  \| \| **Sphericity Correction** \| \| **Sum of Squares** \| \| **df** \| \| **Mean Square** \| \| **F** \| \| **p** \| \| **η²_p_** \| \| \| Experiment \|  \| None \|  \| 6044.85 \|  \| 2 \|  \| 3022.43 \|  \| 1.18 \|  \| 0.312 \|  \| 0.02 \|  \| \|  \|  \| Greenhouse-Geisser \|  \| 6044.85 \|  \| 1.99 \|  \| 3035.08 \|  \| 1.18 \|  \| 0.312 \|  \| 0.02 \|  \| \| Experiment ✻ Cluster \|  \| None \|  \| 15369.84 \|  \| 2 \|  \| 7684.92 \|  \| 2.99 \|  \| 0.054 \|  \| 0.05 \|  \| \|  \|  \| Greenhouse-Geisser \|  \| 15369.84 \|  \| 1.99 \|  \| 7717.08 \|  \| 2.99 \|  \| 0.054 \|  \| 0.05 \|  \| \| Residual \|  \| None \|  \| 298069.90 \|  \| 116 \|  \| 2569.57 \|  \|  \|  \|  \|  \|  \|  \| \|  \|  \| Greenhouse-Geisser \|  \| 298069.90 \|  \| 115.52 \|  \| 2580.32 \|  \|  \|  \|  \|  \|  \|  \| \| Note. Type 3 Sums of Squares \| \| \| \| \| \| \| \| \| \| \| \| \| \| \| \| \|  \| \| \| \| \| \| \| \| \| \| \| \| \| \| \| \|  \| **Complementary analysis Table 2. Between Subjects Effects** \| \| \| \| \| \| \| \| \| \| \| \| \| \| \| --- \| --- \| --- \| --- \| --- \| --- \| --- \| --- \| --- \| --- \| --- \| --- \| --- \| --- \| \|  \|  \|  \|  \|  \|  \|  \|  \|  \|  \|  \|  \|  \|  \| \|  \| \| **Sum of Squares** \| \| **df** \| \| **Mean Square** \| \| **F** \| \| **p** \| \| **η²_p_** \| \| \| Cluster \|  \| 83214.00 \|  \| 1 \|  \| 83214.00 \|  \| 4.36 \|  \| 0.041 \|  \| 0.07 \|  \| \| Residual \|  \| 1107919.52 \|  \| 58 \|  \| 19102.06 \|  \|  \|  \|  \|  \|  \|  \| \| Note. Type 3 Sums of Squares \| \| \| \| \| \| \| \| \| \| \| \| \| \| \|  \| \| \| \| \| \| \| \| \| \| \| \| \| \| | | | | | | | | | | | |

| Complementary analysis Table 3. Post Hoc Comparisons – Cluster and Experiment | | | | | | | | | | | | | | | | | | | | | | | | | | | | | | | | | | | | | | | | | | | | |
| --- | --- | --- | --- | --- | --- | --- | --- | --- | --- | --- | --- | --- | --- | --- | --- | --- | --- | --- | --- | --- | --- | --- | --- | --- | --- | --- | --- | --- | --- | --- | --- | --- | --- | --- | --- | --- | --- | --- | --- | --- | --- | --- | --- | --- |
| **Comparison** | | | | | | | | | | | | | |  | | | | | | | | | | | | | | | | | | | | | | | | | | | | | | |
| **Cluster** | | | |  | | | | **Cluster** | | | | | | **Mean Difference** | | | | | **SE** | | | | | **df** | | | | | | | **t** | | | | | | | | | **p_holm_** | | | | |
| 2 | |  | | - | |  | | 1 | | | | |  | -44.22 | | | |  | 21.19 | | |  | | 58.00 | | | |  | | | -2.09 | | | |  | | | 0.041 | | | | |  |  |
|  | | | | | | | | | | | | | | | | | | | | | | | | | | | | | | | | | | | | | | | | | | | | |
| **Comparison** | | | | | | | | | | | | | | | | | |  | | | | | | | | | | | | | | | | | | | | | | | |  |  |  |
| **Experiment** | | | | | | |  | | | | | **Experiment** | | | | | | **Mean Difference** | | | | **SE** | | | **df** | | | | **t** | | | | | **p_holm_** | | | | | | | | |  |  |
| Personal | | | | |  | | - | |  | | | Reward | | |  | | | 5.33 | |  | | 9.81 | |  | 58.00 | |  | | | 0.54 | |  | | | | 0.665 | | |  | | | | |  |
|  | | | | |  | | - | |  | | | Emotion | | |  | | | -9.10 | |  | | 9.31 | |  | 58.00 | |  | | | -0.98 | |  | | | | 0.665 | | |  | | | | |  |
| Reward | | | | |  | | - | |  | | | Emotion | | |  | | | -14.44 | |  | | 9.42 | |  | 58.00 | |  | | | -1.53 | |  | | | | 0.392 | | |  | | | | |  |
|  | | | | | | | | | | | | | | | | | | | | | | | | | | | | | | | | | | | | | | | | |  |  |  |  |
| Complementary analysis Table 4. Post Hoc Comparisons – Cluster (Bayesian approach) | | | | | | | | | | | | | | | | | | | | | | | | | | | | | | | | | | | | |  |  |  |  |  |  |  |  |
|  |  | |  | | | | | | |  |  | | | | |  |  | | | |  | |  | | |  | | | |  | | |  | | | |  |  |  |  |  |  |  |  |
|  | | |  | | | | | | | | **Prior Odds** | | | | | | **Posterior Odds** | | | | | | **BF_10, U_** | | | | | | | **error %** | | | | | | |  |  |  |  |  |  |  |  |
| 2 |  | | 1 | | | | | | |  | 1.00 | | | | |  | 18.75 | | | |  | | 18.75 | | |  | | | | 0.00 | | |  | | | |  |  |  |  |  |  |  |  |
| Note. The posterior odds have been corrected for multiple testing by fixing to 0.5 the prior probability that the null hypothesis holds across all comparisons (Westfall, Johnson, & Utts, 1997). Individual comparisons are based on the default t-test with a Cauchy (0, r = 1/sqrt(2)) prior. The "U" in the Bayes factor denotes that it is uncorrected. | | | | | | | | | | | | | | | | | | | | | | | | | | | | | | | | | | | | |  |  |  |  |  |  |  |  |
|  | | | | | | | | | | | | | | | | | | | | | | | | | | | | | | | | | | | | |  |  |  |  |  |  |  |  |

**Note 7**

***Cluster profiling: p-values and percentile bootstrap estimate densities***

Exact p-values for each shift function in Personal, Reward and Emotion experiments per cluster are reported in Table S9.

**Table S9. Exact p-values**

|  | **Cluster 1** | | | **Cluster 2** | | |
| --- | --- | --- | --- | --- | --- | --- |
| **Quantiles** | **Personal** | **Reward** | **Emotion** | **Personal** | **Reward** | **Emotion** |
| .005 | .01 | .24 | .03 | .00081 | .61 | .000021 |
| .25 | .002 | .23 | .0003 | .0003 | .62 | .0000086 |
| .5 | .0012 | .18 | .00008 | .000053 | .36 | .0000088 |
| .75 | .001 | .009 | .002 | .0004 | .31 | .0000069 |
| .95 | .002 | .19 | .04 | .0081 | .61 | .00021 |


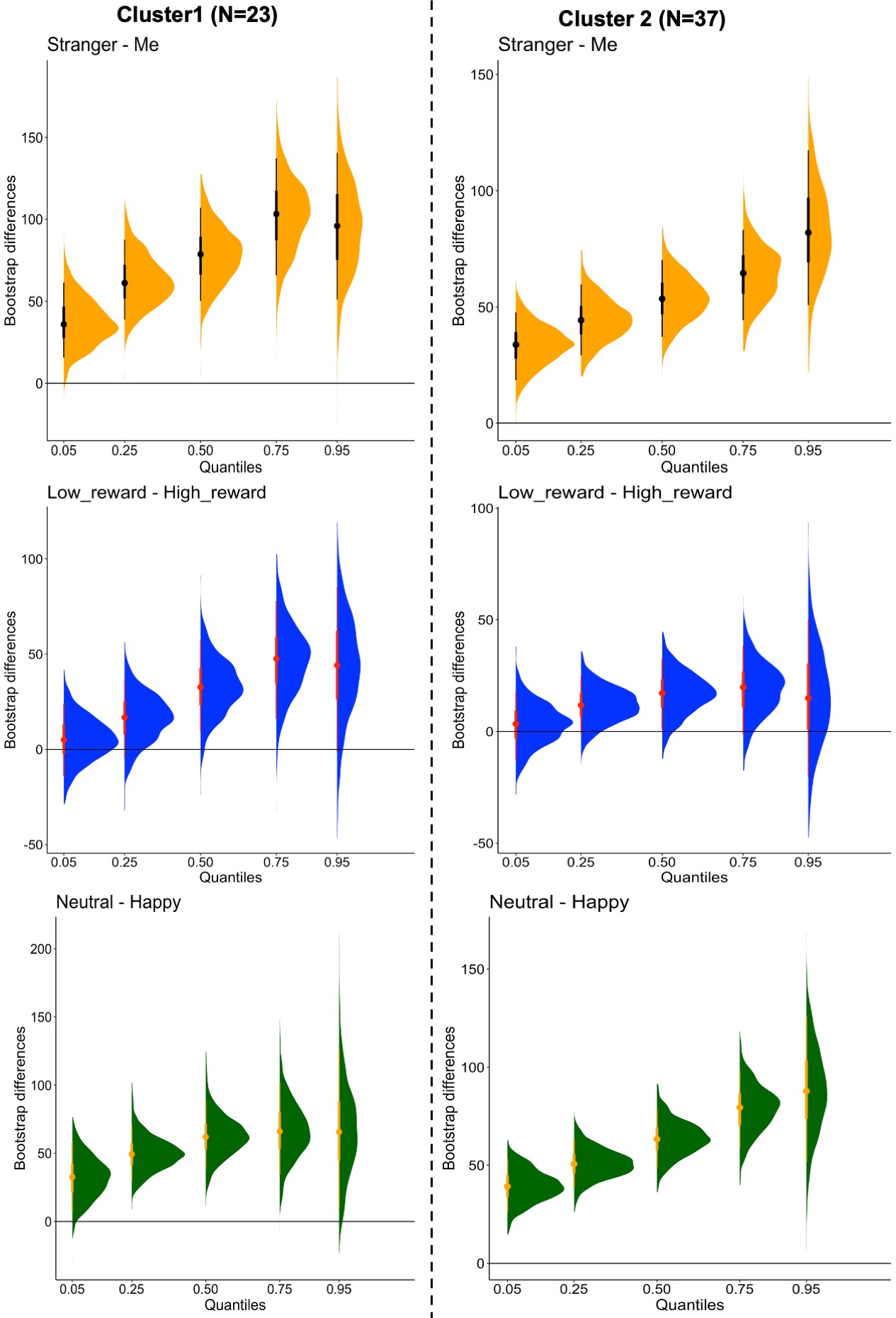


*Figure S11*. Percentile bootstrap estimate densities at each quantile in the Personal, Reward and Emotion experiments per cluster. The distributions of bootstrap estimates of each cluster 20% trimmed means are shown in colour (orange for the Personal, blue for the Reward and green for the Emotion experiment) one for each decile. Along the base of each distribution, the dot marks the mode and the vertical lines mark the 50% and 90% highest density intervals.
